# The structured mRNA element *45ABC* mediates auto‐ and cross‐regulation of RBP45 genes via alternative splicing

**DOI:** 10.1111/tpj.71060

**Published:** 2026-08-18

**Authors:** Maren Reinhardt, Rica Burgardt, Christoph Engel, Maximilian Sack, Zasha Weinberg, Andreas Wachter

**Affiliations:** ^1^ Institute for Molecular Physiology (imP), University of Mainz Hanns‐Dieter‐Hüsch‐Weg 17 55128 Mainz Germany; ^2^ Bioinformatics Group, Department of Computer Science and Interdisciplinary Centre for Bioinformatics Leipzig University Härtelstraße 16‐18 04107 Leipzig Germany; ^3^ Present address: Institute of Computer Science and Institute of Biochemistry and Biotechnology Martin‐Luther University Halle‐Wittenberg 06114 Halle Germany

**Keywords:** alternative splicing, RNA structure, RNA motif, RNA‐binding protein, RBP45, *Arabidopsis thaliana*

## Abstract

Alternative splicing (AS) is a common gene regulatory mechanism involving distinct interactions between *trans*‐acting factors and *cis*‐regulatory elements on the precursor messenger RNA (pre‐mRNA). In this study, we have functionally characterized the structured motif *45ABC*, which is located in the pre‐mRNAs of RNA‐binding protein (RBP) 45 genes in many plant species. Our data revealed that this element mediates a negative auto‐ and cross‐regulatory feedback loop via AS of the three *45ABC*‐containing RBP45 genes in *Arabidopsis thaliana*. We identified a G‐rich stretch within the first stem as a potential RBP45 binding site and observed increased RBP45‐dependent AS upon structural weakening of this pairing element. The second stem includes the alternative 5′ splice site being activated in the presence of RBP45. Based on the known interaction between RBP45 homologs and U1 snRNP components required for 5′ splice site recognition, we propose that RBP45 recruitment to stem I of *45ABC* may induce usage of the alternative 5′ splice site in stem II. The resulting splicing variant is unproductive, thereby diminishing RBP45 expression. Analysing the splicing‐regulatory impact of the three *At*‐RBP45 genes in auto‐ and cross‐regulation and a transcriptome‐wide manner revealed unequal genetic redundancy with a major role of *RBP45B*. Furthermore, phenotypical analysis of single‐ and higher‐order *rbp45* mutants pointed at these genes' functions in controlling primary root growth and flowering time. Taken together, we demonstrated that both sequence and structural features of *45ABC* are critical for proper splicing control, balancing RBP45 expression and functions in plants via a conserved mRNA motif.

## Significance Statement

Functional characterization of a structured mRNA motif present in plant RBP45 genes identified sequence and pairing elements underlying a negative auto‐ and cross‐regulatory expression circuit on the level of alternative splicing. Our study provides a rationale for the evolutionary conservation of this RNA element, which allows balancing levels and functions of RBP45 proteins as a requirement for proper plant development.

## INTRODUCTION

During their life cycle, plants need to continuously adapt to environmental fluctuations, involving precise genetic control to fine‐tune cellular and developmental processes. A critical layer of this regulation is the splicing of precursor messenger RNAs (pre‐mRNAs), a co‐ or post‐transcriptional process that can occur in either a constitutive or alternative manner. While during constitutive splicing the same exon sequences are incorporated in the same order in the mature mRNA, multiple mRNA isoforms are generated by alternative splicing (AS) due to differential 5′ and 3′ splice site selection. For *Arabidopsis thaliana*, an average of 4.4 transcript isoforms per gene was reported to be generated by AS and the usage of alternative transcript start and end sites (Zhang et al., 2022). Numerous studies have provided evidence that plants exploit the potential of AS in fine‐tuning gene expression to modulate development, responses to stress and adaptation to environmental changes (Alhabsi et al., 2025; Reddy et al., 2013; Staiger & Brown, 2013). AS can expand both transcriptomic and proteomic diversity. A substantial proportion of the AS variants is likely not translated into proteins but instead may influence gene expression by altering other processes such as mRNA stability and localization. One widespread mechanism represents the coupling of AS and nonsense‐mediated decay (NMD), which causes turnover of non‐productive isoforms and thereby allows quantitative gene control (Drechsel et al., 2013; Kalyna et al., 2012).

Eukaryotic pre‐mRNA splicing is regulated by the interplay of *cis*‐regulatory elements, specific sequences and structures within the target RNAs and *trans*‐acting factors, including RNA and protein molecules. The splicing regulatory proteins include two major classes: the serine/arginine‐rich (SR) proteins and heterogeneous nuclear ribonucleoprotein (hnRNP) proteins. Originally, SR and hnRNP proteins, respectively, have been mainly considered as activators and repressors of splicing sites; however, it is now clear that their action is highly context‐dependent and shaped by parameters such as the binding location and the presence of other regulatory factors and elements (Fu & Ares, 2014; Reddy et al., 2013). Among the diverse group of hnRNP proteins from *A. thaliana* (Wachter et al., 2012), the glycine‐rich RNA‐binding proteins (RBPs) GRP7 and GRP8 (Streitner et al., 2012) and polypyrimidine tract‐binding proteins (PTBs; Rühl et al., 2012) have been intensively studied and linked to AS. Both GRPs and PTBs are part of auto‐ and cross‐regulatory circuits, triggering formation of NMD‐targeted splicing variants from their own and the homologs' pre‐mRNAs when the levels of the respective proteins are elevated. This type of balancing mechanism is common among splicing regulators and other RBPs in plants and other organisms (Müller‐McNicoll et al., 2019; Reddy et al., 2013; Wachter et al., 2012).

The RBP45 family also belongs to the hnRNP proteins and comprises in *A. thaliana* the four members RBP45A, RBP45B, RBP45C and RBP45D, each of which contains three RNA recognition motifs (RRMs; Lorković et al., 2000). So far, their specific functions and in particular their possible role in AS regulation remain largely unresolved. Previous work has shown that RBP45D interacts with pre‐mRNA‐processing factor 39a (PRP39a), a component of the U1 small nuclear ribonucleoprotein (snRNP), suggesting a function in 5′ splice site selection (Chang et al., 2022). Moreover, RBP45D was found to promote flowering (Wang et al., 2022). *In vitro* interaction studies with RBP45B confirmed its RNA binding (Peal et al., 2011), and RBP45 proteins were found to associate with proteins involved in various aspects of RNA processing, including U1 snRNP components (Mangilet et al., 2024), cap‐binding protein 20 (CBP20) and the poly(A)‐binding protein PAB8 (Muthuramalingam et al., 2016) and the RNA helicase up‐frameshift 1 (UPF1; Chicois et al., 2018; Sulkowska et al., 2020). Recently, the presence of an evolutionary conserved structured RNA (strucRNA), *45ABC*, was reported for the pre‐mRNAs of *RBP45A*, *RBP45B* and *RBP45C*, but not *RBP45D* (Sack et al., 2025).

In addition to *trans*‐acting factors, RNA sequence and structural motifs play a crucial role in facilitating exon and intron definition (Buratti & Baralle, 2004; Georgakopoulos‐Soares et al., 2022; Ullah et al., 2018). For instance, local RNA structures can alter the accessibility of *cis*‐regulatory elements, thereby either hindering or enhancing spliceosomal assembly (Shepard & Hertel, 2008). The ability of RNA to form dynamic structures gives rise to a structural complexity that can be responsive to various external factors such as temperature (Chung et al., 2020), subcellular localization (Sun et al., 2019) and interactions with RBPs (Gosai et al., 2015) or metabolite ligands (Steinert et al., 2017). RNA secondary structures have been shown to influence diverse steps of gene expression such as AS (Wachter, 2014), RNA localization (Fernández‐Moya et al., 2021) and RNA stability (Fischer et al., 2020; Goodarzi et al., 2012). While AS regulation through strucRNAs is well documented in animals (Raker et al., 2009; Shepard & Hertel, 2008), few such instances have been reported in plants. One example is the thiamine pyrophosphate (TPP)‐binding riboswitch, a conserved RNA structural element found across all three domains of life, including fungi and plants, but being absent from animals (Bocobza et al., 2007; Sudarsan et al., 2003; Wachter et al., 2007). Upon binding of its ligand TPP, the riboswitch's conformation changes, demasking an alternative splice site and thereby altering the splicing pattern and downregulating the thiamin biosynthesis gene *THIC* in a negative feedback loop. Another example for an RNA structure that is conserved in plants and involved in AS control is the plant 5S ribosomal RNA structural mimic (P5SM; Hammond et al., 2009). It interacts with a ribosomal protein to modulate AS of a transcription factor pre‐mRNA, adjusting 5S rRNA production to the level of its binding partner L5 (Hammond et al., 2009).

In this study, we have characterized the AS‐regulatory function of a structured mRNA element that was recently found in a bioinformatic survey of the non‐coding regions of plant pre‐mRNAs (Sack et al., 2025). The strucRNA *45ABC* consists of two hairpins, which are highly conserved among homologs of *RBP45A*, *RBP45B* and *RBP45C* from monocots and dicots. It consistently overlaps with a cassette exon (CE), the skipping of which generates a splicing variant encoding the full‐length RBP45 protein, while its inclusion introduces a premature termination codon (PTC) and triggers degradation via NMD (Sack et al., 2025). Using splicing reporters based on the three *At*‐RBP45 genes, we revealed negative feedback regulation of their pre‐mRNAs via AS. Disruption of the motif's first hairpin resulted in a strong increase in the CE variant, while compensatory mutations restored the authentic splicing output. Further mutational studies identified within this stem I a G‐rich sequence that is required for efficient CE inclusion and is proposed to serve as an RBP45 binding site. Moreover, we have established in *A. thaliana* individual overexpression (OE) lines as well as single and higher‐order *rbp45* knockout mutants to examine these genes' regulatory crosstalk as well as functions in global AS control and plant growth. Transcriptome‐wide analyses provided evidence that out of these three RBP45 genes from *A. thaliana* only *RBP45B* plays a broader role in AS control beyond the cross‐regulatory circuit. Furthermore, all of the tested loss‐of‐function mutants exhibited significantly shorter primary roots compared to wild‐type (WT) plants. In summary, our study has unravelled novel functions of RBP45 genes in *A. thaliana* and identified the strucRNA *45ABC* as a mediator of an AS‐based gene regulatory circuit that achieves specificity due to a combination of sequence and structural features.

## RESULTS

### The *45ABC* motif overlaps with AS sites of *RBP45* genes

**Figure 1:**
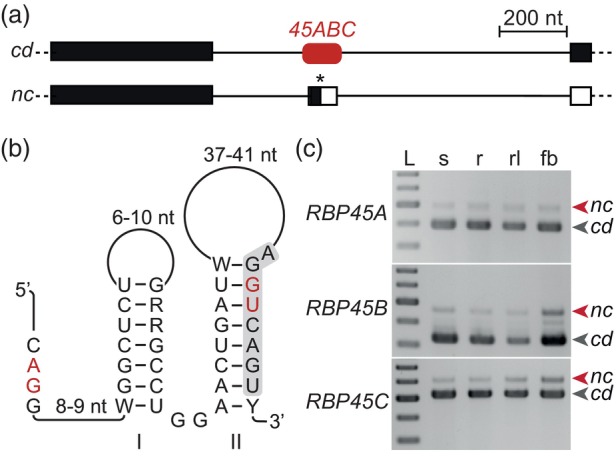
Alternative splicing of *RBP45A*, *RBP45B* and *RBP45C* genes is associated with the structured element *45ABC*. (a) Schematic depiction of alternatively spliced region of *RBP45A* (*AT5G54900*), *RBP45B* (*AT1G11650*) and *RBP45C* (*AT4G27000*) giving rise to coding (*cd*) and non‐coding (*nc*) isoforms with the cassette exon (CE) encompassed by the strucRNA *45ABC* (rounded red rectangle). Premature termination codon (PTC) indicated by asterisk; exons, introns, CDS and untranslated regions (UTRs) are depicted by boxes, lines, black and white shading, respectively. (b) Predicted secondary structure of strucRNA *45ABC* based on the consensus sequence from the three representatives in *Arabidopsis thaliana*. Red letters show alternative splice sites used for CE inclusion. Letters indicate nucleotides (nt); W, A or U; R, A or G; Y, C or U; lines show variable‐length regions. Grey shading marks U1 snRNP‐binding region. Stems I and II are labelled. (c) Alternative splicing (AS) patterns of RBP45 genes based on RT‐PCR products of samples from 11‐day‐old whole seedlings (s), roots of 7‐day‐old seedlings (r), rosette leaves (rl) and flower buds (fb). Binding sites of corresponding primer pairs indicated in Figure S2a. L, size marker in 100 bp increments from 0.3 to 0.7 kb.

A previous bioinformatic analysis of non‐coding regions in available plant genome data revealed several conserved strucRNA candidates that overlap with alternative splice sites (Sack et al., 2025). One promising candidate from this study had previously been identified as a conserved intronic element in RBP genes from angiosperms (Burgess & Freeling, 2014); however, its functional role remained unknown. Since it is associated with *RBP45A* (*AT5G54900*), *RBP45B* (*AT1G11650*) and *RBP45C* (*AT4G27000*) from *A. thaliana*, it was named *45ABC* motif. In the data sets analysed, the motif has 448 matches in genes from 112 mono‐ and dicotyledonous species that are annotated as *RBP45* homologs or are related to *RBP45* (Data Set S1). Interestingly, for all *RBP45* homologs, *45ABC* is positioned in a long intron and encompasses a CE (Figure 1a). Moreover, the two predicted hairpin loops of the motif are in the vicinity of or include the alternative splice sites (Figure 1b; Figure S1a), with the alternative 3′ splice site being located ~10 nt upstream of stem I and the alternative 5′ splice site and the U1 snRNP‐binding motif (consensus sequence: AG/GUAAGU according to Sheth et al. (2006)) being embedded in stem II. Analysis of the *RBP45* transcripts revealed the generation of two major AS variants: Skipping of the CE leads to an isoform (*cd*) that is predicted to encode the full‐length protein. CE inclusion introduces a PTC and a long 3′ untranslated region (UTR), making this isoform (*nc*) probably unproductive. Accordingly, NMD targeting of the CE inclusion variants from these three *RBP45* genes from *A. thaliana* was experimentally confirmed (Drechsel et al., 2013; Sack et al., 2025). Reverse transcription‐polymerase chain reaction analysis of *RBP45* splicing in different tissues revealed an overall similar splicing output, with the coding variant being the predominant amplification product in all samples (Figure 1c; Figure S1b).

### Arabidopsis RBP45 homologs regulate AS of their own pre‐mRNAs

**Figure 2:**
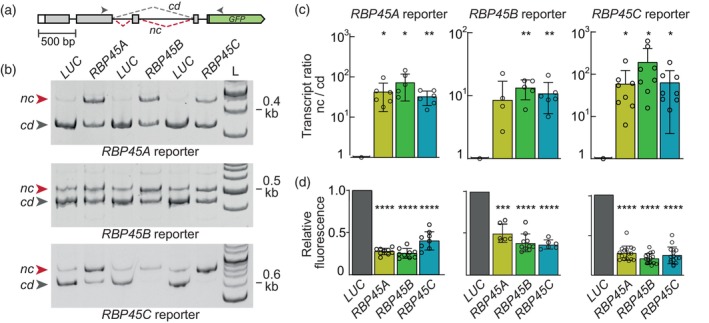
RBP45A, RBP45B and RBP45C can induce inclusion of the cassette exon overlapping with the *45ABC* strucRNA. (a) Design of splicing reporter constructs for RBP45 genes (display based on *RBP45C*) with boxes and lines representing exons and introns, respectively; arrowheads show the approximate binding sites of primers used for co‐amplification PCR shown in (b). (b) RT‐PCR products of splicing variants from reporters based on *RBP45A* (top), *RBP45B* (middle) and *RBP45C* (bottom) upon transient expression in *Nicotiana benthamiana* leaves co‐infiltrated with luciferase (*LUC*) or indicated CDS constructs were resolved via native PAGE. L: size marker, in 100 bp increments up to 1 kb. (c, d) Quantification of reporter output based on alternative splicing (AS) ratios of RT‐PCR products analysed via Bioanalyzer (c) and green fluorescent protein (GFP) fluorescence (d). Bars indicate mean values, error bars show standard deviations, and open circles represent individual data points. AS ratio and fluorescence of LUC samples each was set to 1. Asterisks indicate significant differences compared to LUC control (one sample t‐test, *P < 0.05, **P < 0.01, ***P < 0.001, ****P < 0.0001).

The association of the strucRNA *45ABC* with the alternative splice sites in the corresponding pre‐mRNAs indicated a functional relationship. To explore a potential regulatory circuit, we designed splicing reporters based on the genomic DNAs of *RBP45A, RBP45B* and *RBP45C* spanning the region from the 5′ UTR to the beginning of the second exon downstream of the CE (Figure 2a). These reporters included an in‐frame fusion of the CDS of green fluorescent protein (GFP), resulting in a fluorescent fusion protein upon reporter splicing to the *cd* variant (Figure 2a). Infiltration assays in *Nicotiana benthamiana* leaves were used to examine reporter splicing, in the presence of co‐expressed CDS constructs of *RBP45A*, *RBP45B* or *RBP45C* compared to a control construct containing the CDS of luciferase (*LUC*). In the *LUC* samples, all reporters showed splicing into both transcript variants, with *cd* being the predominant one (Figure 2b; Figure S2). Co‐expression of any of the *RBP45* CDS constructs shifted splicing patterns towards the respective *nc* isoform, with a ~10‐fold to ~100‐fold increase in the *nc*/*cd* ratio for the different reporter constructs (Figure 2b,c; Figure S2). This shift in AS towards the *nc* variants correlated with the reduced fluorescence levels of all reporters to less than 50% in the presence of the CDS constructs of *RBP45A*, *RBP45B* or *RBP45C* (Figure 2d). Furthermore, each reporter was regulated by the three RBP45 proteins to a similar extent in this transient assay system.

**Figure 3:**
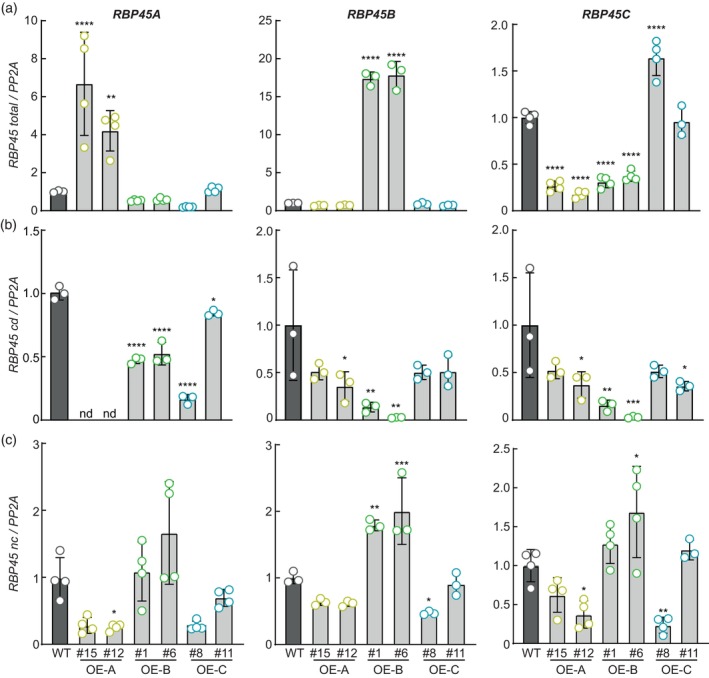
Analysis of negative auto‐ and cross‐regulation in RBP45 overexpression (OE) lines unveils a major function of *RBP45B* in splicing control. Quantitative PCR analysis of total (a), endogenous coding (b) and endogenous non‐coding (c) *RBP45* transcript levels in 11‐day‐old *RBP45 OE Arabidopsis thaliana* seedlings. All values are expressed relative to reference transcript *PP2A* and normalized to the respective wild‐type (WT) mean. Circles represent individual data points from biological replicates and standard deviations are depicted. Asterisks indicate significant change compared to WT (one‐way anova followed by Dunnett's multiple comparisons test, *P < 0.05, **P < 0.01, ***P < 0.001, ****P < 0.0001); nd, not determined.

To assess this regulatory circuit in its native context, we modulated *RBP45A*, *RBP45B* and *RBP45C* expression in stably transformed *A. thaliana* plants. This was accomplished either through their OE (*35S:RBP45CDS*) or by CRISPR/Cas9‐mediated knockout of one, two or all three corresponding *RBP45* genes (Figure S3). Analysis of total *RBP45* transcript levels confirmed the overaccumulation of *RBP45* mRNAs in the corresponding OE lines (Figure 3a). Notably, the *OE‐B* lines showed the strongest extent of OE with ~17 times higher *RBP45* amounts compared to WT. The two independent *OE‐A* lines showed an only ~5‐fold OE, while for *RBP45C* only one OE line with a less than 2‐fold increase in *RBP45C* could be obtained. The other *RBP45C*‐OE line probably showed silencing as the level of the coding *RBP45C* transcript from the endogenous locus was decreased and total *RBP45C* levels were WT‐like (Figure 3a,b). In general, an increase in the total transcript levels of one of the three *RBP45* genes led to a downregulation of the other two. This effect was particularly pronounced for the paralogs *RBP45A* and *RBP45C*. Furthermore, coding levels of the endogenous genes decreased consistently for all OE lines (Figure 3b). Interestingly, an upregulation of the non‐coding transcript variants was only observed in the case of the *OE‐B* lines, and this effect was consistent among all three genes (Figure 3c), indicating a unique role of RBP45B in AS control. In contrast, OE of *RBP45A* or *RBP45C* resulted in downregulation of the coding and non‐coding AS variants from the homologous endogenous genes. This decrease of the non‐coding variants in the *RBP45A*‐ and *RBP45C*‐OE lines may be a consequence of their reduced levels of *RBP45B.cd*. Accordingly, the non‐coding variants of all three genes may predominantly arise through RBP45B activity in *A. thaliana*. Given that upon transient expression in *N. benthamiana* all three RBP45 proteins similarly regulated reporter splicing (Figure 2), it seems likely that the specificity observed for RBP45B in the stable mutant lines is at least not solely caused by protein features but might also involve other aspects such as its expression and regulation.

**Figure 4:**
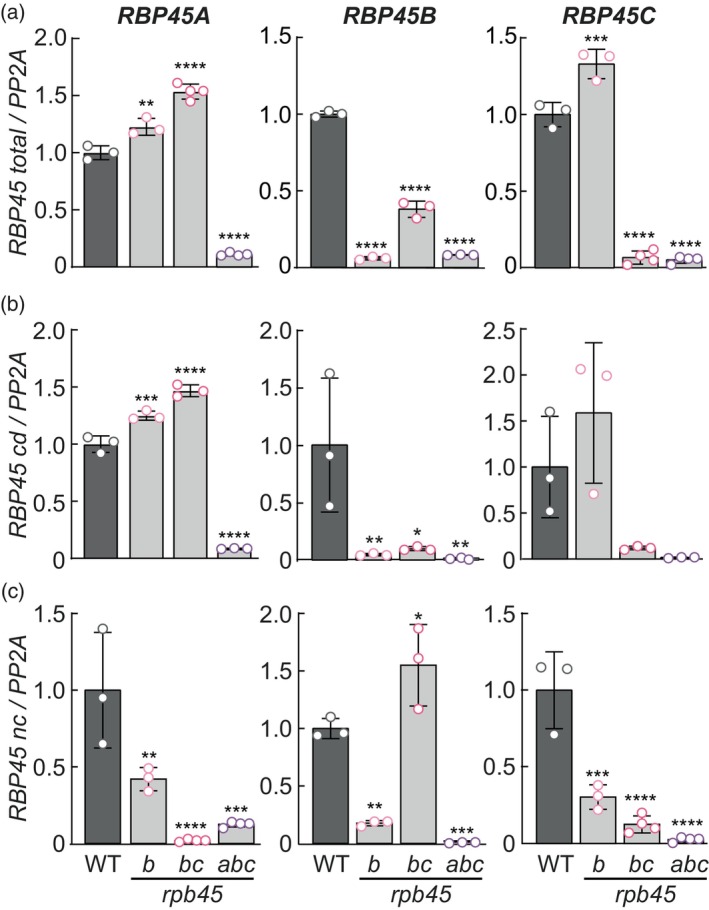
RBP45 knockouts reveal the crosstalk and hierarchy in RBP45‐mediated splicing regulation. Quantitative PCR analysis of total (a), coding (b) and non‐coding (c) RBP45 transcript levels in 11‐day‐old *Arabidopsis thaliana* seedlings in *rbp45* single and higher‐order mutants. All values are expressed relative to *PP2A* and normalized to the respective wild‐type (WT) mean. Circles represent individual data points from biological replicates and standard deviations are depicted. Asterisks indicate significant change compared to WT (one‐way anova followed by Dunnett's multiple comparisons test, *P < 0.05, **P < 0.01, ***P < 0.001, ****P < 0.0001).

To complement these findings and further dissect the roles of *RBP45A*, *RBP45B* and *RBP45C* in AS regulation, we also analysed transcript levels and splicing patterns of the *RBP45* genes in the three mutant lines generated via CRISPR/Cas9 mutagenesis: *rbp45b*, *rbp45bc* and *rbp45abc* (Figure S3). As the corresponding mutations give rise to PTCs at early positions within the CDS, these mutants are considered to be knockout lines. Total transcript levels of the respective mutated genes were significantly reduced compared to those in WT seedlings, and a slight overaccumulation of the non‐targeted homolog(s) was seen (Figure 4a). In the *rbp45b* mutant, a decrease of non‐coding isoforms was observed for all three *RBP45* genes, whereas the coding transcript variants of *RBP45A* and *RBP45C* showed an opposite trend (Figure 4b,c). This further supports that *RBP45B* is a major AS regulator for all three *RBP45* genes. Interestingly, knocking out *rbp45c* in addition to *RBP45B* in the double mutant *rbp45bc* restored levels of non‐coding *RBP45B* transcript and caused an additional decrease and increase, respectively, of the non‐coding and coding *RBP45A* variant (Figure 4b,c). Accordingly, the expression of *RBP45C* and *RBP45A* is tightly linked. Furthermore, the low levels of non‐coding variants for all three RBP45 genes in the triple mutant suggested that the cross‐regulatory AS circuit might be restricted to this set of genes.

### Structural and sequence features of *45ABC* contribute to AS control of RBP45 genes

**Figure 5:**
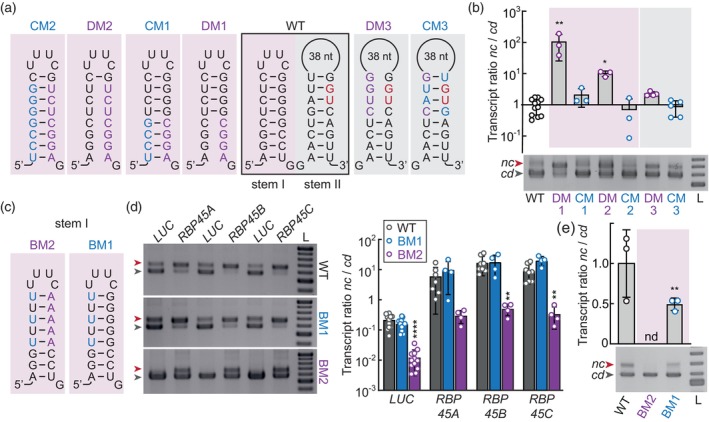
Both structural and sequence features of *45ABC* are required for proper cassette exon splicing. (a) Schematic representation of the base‐pairing potential of *45ABC* in its wild‐type (WT) form of *RBP45C*, as well as disruptive (DM) and compensatory (CM) mutations. Stem I and II areas shaded in pink and grey, respectively. Alternative 5′ splice site is indicated in red letters and mutated nucleotides are shown in purple (DM) or blue (CM). (b) Bioanalyzer quantification of WT and mutant *45ABC* reporter alternative splicing (AS) in 11‐day‐old stably transformed *Arabidopsis thaliana* seedlings. Reporter constructs based on *RBP45C* sequence. Upper part shows reporter AS ratios with mean value of WT reporter set to 1. Bars show mean values and individual data points correspond to independent transformant lines. Standard deviations are depicted, and asterisks indicate significant change compared to the WT reporter (one‐way anova followed by Dunnett's multiple comparisons test, *P < 0.05, **P < 0.01). Lower part shows agarose gel analyses of representative samples with grey and red arrowheads indicating RT‐PCR bands from coding and non‐coding splice variants, respectively. L: size marker, from bottom to top: 0.5–0.8 kb, in 0.1 kb increments. (c) Schematic representation of *45ABC's* stem I for the potential binding motif mutations (BM1, BM2) in the context of the *RBP45C* splicing reporter. (d) RT‐PCR analysis of splicing variants derived from *RBP45C* WT, BM1 and BM2 reporter upon co‐expression in *Nicotiana benthamiana* leaves with luciferase (*LUC*) or indicated CDS constructs. L: size marker, from bottom to top: 0.4–1.0 kb in 0.1 kb increments. Log‐scale quantification depicts mean values with standard deviation; open circles represent individual data points. Asterisks indicate significant differences compared to the WT construct (two‐way anova followed by Dunnett's multiple comparisons test; **P < 0.01, ****P < 0.0001). (e) AS analysis of WT and BM mutant *45ABC* reporter in 11‐day‐old stably transformed *A. thaliana* seedlings. Details as described for (b); nd, not determined as *nc* variant was undetectable (one sample t‐test, **P < 0.01).

Given the association between the cassette exon and the *45ABC* motif, we next addressed the possible functional role of this strucRNA in controlling the AS output. To this end, we created the following mutations in the *45ABC* motif in the context of the *RBP45C* splicing reporter, which showed the largest dynamic range of regulation in the transient assays (Figure 2): The disruptive mutants DM1 and DM3 in stem I and stem II, respectively, each involving the exchange of four nucleotides to eliminate base‐pairing and destabilize the corresponding pairing elements (Figure 5a). At the same time, we ensured that the alternative 5′ splice site within stem II was preserved at its authentic location. To further examine the contribution of the stem I structure, we designed an additional mutant DM2, containing three further nucleotide substitutions and resulting in an almost complete loss of the pairing potential within this part of the element. To exclude that any changes observed for the DMs are due to a change in sequence and not the consequence of the structural disturbance, we created compensatory mutations CM1, CM2 and CM3 that are expected to restore the original structure in the context of the new sequence (Figure 5a). Accordingly, if the splicing outcome is primarily dependent on structural features of the mutated regions of the *45ABC* motif, the splicing pattern of the DM and CM constructs, respectively, should be disrupted and restored in comparison to the WT reporter.

We initially tested these splicing reporters upon transient expression in *N. benthamiana* leaves and observed that the disruptive mutants DM1 and DM2 in stem I resulted in almost exclusive splicing to the *nc* isoform, even without RBP45 CDS co‐expression (Figure S4a). For the stem II‐affecting DM3, only a partial splicing shift towards the *nc* variant was seen, and this effect was further enhanced upon RBP45 co‐expression. All compensatory mutations reverted this splicing pattern by causing a relative increase of the *cd* variant; however, in comparison to the WT construct, CM1 and CM2 did not fully compensate while CM3 overcompensated. In the case of the stem I mutants, the response to RBP45 co‐expression was almost completely lost and restored, respectively, for the DM and CM constructs (Figure S4b,c). As the transient analysis in *N. benthamiana* leaves involves artificially high expression, we next tested splicing of the various reporter constructs in the endogenous context. In stably transformed *A. thaliana* lines, DM1 also led to a significant shift towards the *nc* isoform (Figure 5b). In contrast to the transient system, here CM1 was able to restore the splicing pattern to a WT‐like AS ratio. The more extensive mutation DM2 in stem I also resulted in a shift towards the *nc* isoform, although this effect was quantitatively less pronounced than in the case of DM1. Nevertheless, CM2 also fully restored the WT splicing pattern, being in line with the critical role of the stem I structure. The stem II‐targeting DM3 showed only a slight increase in the *nc* /*cd* ratio, in agreement with the findings from the transient assays. Still, the CM3 mutation reverted this minor splicing shift back to WT levels, suggesting that stem II can also contribute to splice site selection (Figure 5b). Collectively, these results from transient and stable transformation experiments demonstrated that the structure of *45ABC*, particularly of stem I, plays a critical role in modulating the AS outcome of the corresponding RBP45 genes.

We hypothesized that the base‐pairing within stem I may affect the splicing outcome by controlling the accessibility of a region required for RBP45 recruitment. A prior study revealed that recombinantly expressed RBP45 protein from *Nicotiana plumbaginifolia* exhibits *in vitro* binding to poly(U) and poly(G) ribohomopolymers (Lorković et al., 2000). Interestingly, a consecutive stretch of guanines is present in stem I of *45ABC* in the case of *RBP45C*. For *RBP45A* and *RBP45B*, one of the guanines is replaced by an adenine (Figure S1a). Analysis of all available instances of this motif identified in this region the purine‐rich sequence RGRG (Sack et al., 2025). To experimentally test the relevance of the G‐stretch within stem I, additional mutations were generated in the context of the *RBP45C* splicing reporter. In the binding mutant BM2, the consecutive guanines were substituted with adenines (Figure 5c). Additionally, three C nucleobases on the complementary strand were mutated to Us to preserve the pairing potential. As a control, we also designed the mutant BM1, in which the complementary poly(U) stretch was included while the G‐stretch and the pairing potential were preserved (Figure 5c). Upon transient co‐expression with the *LUC* control in *N. benthamiana* leaves, BM1 displayed a WT‐like splicing pattern, whereas BM2 exhibited a near‐complete loss of splicing to the CE‐containing variant (Figure 5d). In line with the increased splicing of BM2 to the coding variant, this mutant resulted in higher reporter fluorescence than the WT construct (Figure S4c). When co‐transformed with the CDS constructs of any of the three RBP45 homologs, BM2 still showed an AS response; however, the AS ratio *nc*/*cd* for this mutant was markedly lower compared to the WT and the BM1 construct (Figure 5d). This responsiveness of the BM mutants could also be seen at the protein level of the reporters, with reduced fluorescence upon RBP45 co‐expression (Figure S4d). The observation that splicing of the BM2 mutant reporter is still responsive to RBP45 co‐expression might be explained by massive accumulation of the corresponding RBP45 proteins and possibly less specific RNA binding in the *N. benthamiana* system. We therefore tested AS of the BM reporter constructs also in stably transformed *A. thaliana* lines. Here, the BM2 mutant showed exclusive splicing to the coding variant (Figure 5e), underlining the critical role of the G‐stretch in proper AS regulation. The more WT‐like splicing pattern of the BM1 construct confirmed that the altered AS pattern for BM2 was not the consequence of introducing the complementary U stretch.

Taken together, both sequence and structural features of *45ABC* can contribute to *cis* regulation of the corresponding AS events. The G‐rich stem in stem I may function in recruitment of RBP45 and/or other splicing regulators as a requirement for CE inclusion. Such a model is also in line with the observation that DM2, which lacks the G‐stretch, showed a weaker AS change than DM1, where said G‐stretch is preserved; despite a more pronounced structural impairment in stem I in the former mutant.

### Misexpression of RBP45 can reduce primary root length and alter flowering time

**Figure 6:**
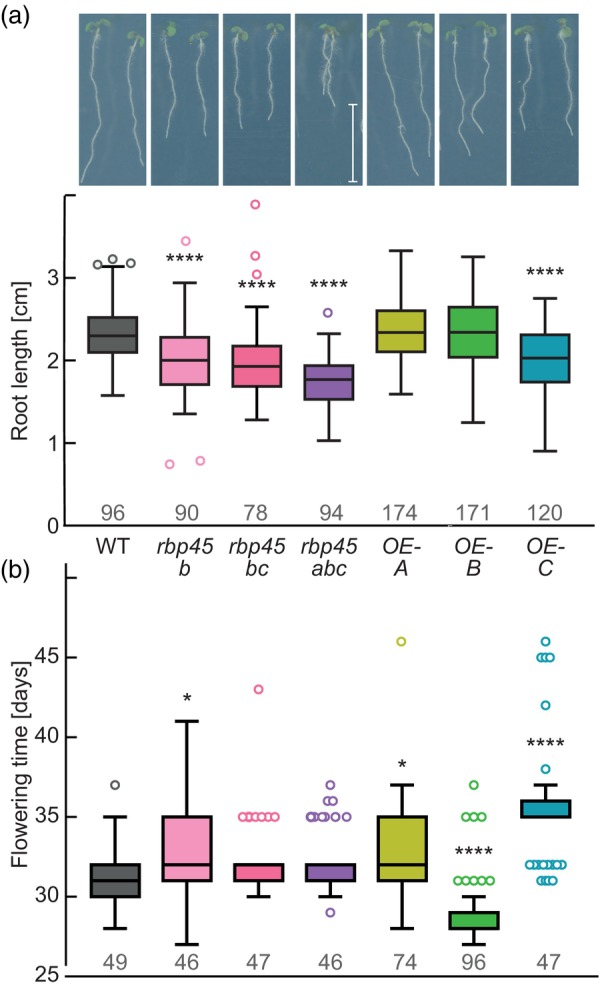
Root length and flowering time are altered upon RBP45 misexpression. (a) Primary root lengths of 7‐day‐old *Arabidopsis thaliana* wild‐type (WT) and RBP45 misexpression lines depicted by representative pictures and mean values with standard deviations. White scale bar corresponds to 1 cm and numbers of analysed seedlings from three independent experiments are indicated. Asterisks indicate significant change compared to WT (one‐way anova followed by Dunnett's multiple comparisons test; ****P < 0.0001). (b) Flowering time in days after sowing. Lines, display details and statistical analysis (*P < 0.05, ****P < 0.0001) as described for (a).

To gain insights into the biological relevance of the RBP45 genes, we compared different developmental aspects between our misexpression lines and WT plants. A previous study reported reduced primary root lengths for *rbp45d* mutants, while no such phenotype was seen for *rbp45a* and *rbp45c* T‐DNA insertion lines (Huang et al., 2022; Muthuramalingam et al., 2016). Determining root lengths of the mutant lines revealed that the loss of *RBP45B* also resulted in shorter roots, and this phenotype was even more pronounced in the corresponding double and triple mutants (Figure 6a). A diminished root length was also observed for the *OE‐C* line, whereas the two other OE lines showed WT‐like root elongation. To test whether the altered root phenotype of the corresponding lines might be a consequence of diminished seed size, we measured the seed surface area of the mutant lines and different batches of WT seeds. The variation observed among the mutants and different batches of WT seeds was in a similar range (Figure S5a,b), indicating that the altered root length can at least not solely be explained by varying seed filling and resource availability.

Inspection of rosette development showed WT‐like growth for all the analysed mutants except *OE‐C* that displayed on average a slightly reduced rosette diameter (Figure S5c,d). This mutant also flowered later than the other genotypes (Figure 6b), which might relate to a general delay in development. Interestingly, the *OE‐B* line started flowering at a significantly earlier time point than the WT, while the *OE‐A* and *rbp45b* plants displayed slightly delayed flowering. Based on similar numbers of rosette leaves at the onset of flowering, all lines were then in a comparable developmental stage (Figure S5e). In line with our observations, Muthuramalingam et al. (2016) also observed delayed flowering of *rbp45b* mutant plants. Moreover, previous studies of *rbp45d* mutants (Huang et al., 2022) demonstrated later flowering as well, pointing to a complex role of RBP45 family members in this developmental context. Accordingly, balancing expression of RBP45 genes is expected to be critical for their functions in certain aspects of development.

### RBP45B is the major regulator of AS among the three RBP45 paralogs

**Figure 7:**
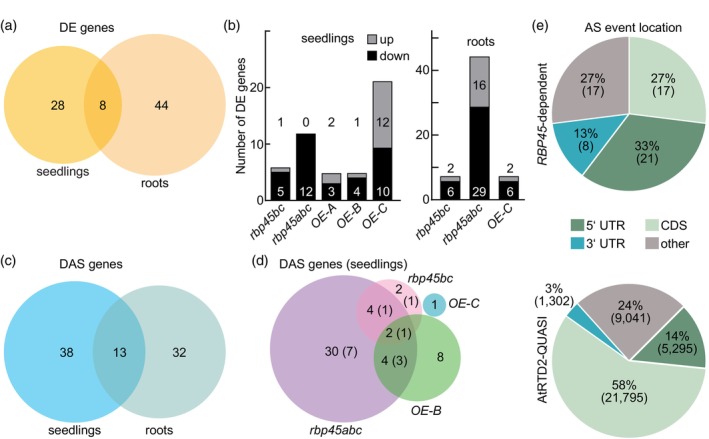
RBP45 misexpression affects expression and alternative splicing (AS) of relatively few genes in *Arabidopsis thaliana*. (a, b) Differential gene expression in *rbp45* mutants compared to wild‐type (WT) for 10‐day‐old whole seedlings or roots from 7‐day‐old plants. Venn diagram (a) shows numbers of combined significant genes, while bar plot (b) provides line‐wise comparisons and direction of changes. (c, d) Genes showing differential AS for samples as defined in (a, b). Venn diagram of DAS genes cumulated for seedling and root samples (c) or for individual mutant comparisons in seedlings (d) are displayed. Numbers correspond to DAS genes, with the number in parenthesis in (d) indicating the overlap with the root data. (e) Positioning of DAS events (RBP45‐dependent, top) compared to all events (AtRTD2‐QUASI, bottom) in proportions and total numbers (in parentheses). Based on their location relative to translational start and stop sites, events were assigned to the 5′ untranslated region (UTR), CDS or 3′ UTR; ‘other’ refers to events that were overlapping or could not be located, as further described in Figure S11c and the method section.

Since we observed that the three RBP45 genes are subject to AS‐mediated auto‐ and cross‐regulation, we next investigated to which extent the RBP45 genes can influence gene expression and AS at the whole transcriptome level. Therefore, RNA sequencing analyses were performed using *A. thaliana* seedling samples of the higher‐order mutants *rbp45bc* and *rbp45abc* as well as the constitutive OE lines for *RBP45A*, *RBP45B* and *RBP45C*. Based on the altered root phenotype, we also analysed root tissue of 7‐day‐old WT seedlings and mutants with shorter primary roots, namely *rbp45bc*, *rbp45abc* and *OE‐C*. Data were analysed via the 3D RNA‐seq pipeline (Guo et al., 2021), resulting for both datasets in the detection of over 16 000 genes that were expressed in at least one sample type (Figures S6 and S7). The gene expression analysis revealed rather few changes, with 36 and 52 differentially expressed (DE) genes, respectively, in the whole seedlings and root samples (Figure 7a). Only eight of the DE genes were shared between the two datasets, indicating the involvement of tissue‐specific regulation. Most of the DE genes were downregulated in the mutants, both for whole seedlings and roots (Figure 7b; Data Set S2) and with the largest number of DE genes being detected in *rbp45abc* roots. In line with this, the triple mutant showed the most pronounced reduction in root length. Among the DE genes was a *copia‐like retrotransposon* (*AT5G35935*), whose function is uncharacterized yet and which was consistently downregulated in both seedlings and roots in all RBP45 misexpression lines relative to WT samples (Figure S8). Given that the alteration in the transcript level of this retrotransposon showed no correlation with RBP45 expression level in the mutants, we concluded that this change is probably not directly linked to RBP45 function.

The separate DE analyses for seedling and root samples suggested that only a few genes are regulated in an *RBP45*‐dependent manner. We next extended our analysis to the line‐wise comparison of tissue‐specific expression patterns, which might differ between the mutants and the WT and could have been missed in the previous comparisons among seedling or root samples due to small quantitative effects. As we used in our study a different number of replicates for the seedling and root samples, we first performed pair‐wise comparisons for the WT and three types of mutants (Figure S9a–d). The comparison of the replicates and genotypes gave consistent results, with an overlap of 4277 (68.4%) DE genes that show differential expression in seedlings and roots of both WT and the two knockout mutant backgrounds *rbp45bc* and *rbp45abc* (Figure S9e). Additionally, including the *OE‐C* line reduced the number of common DE genes only slightly (Figure S9f; 4100 genes, 63.2%), thus representing a robust signature of tissue‐specific gene expression. A GO term analysis for this gene set revealed, among others, an enrichment of photosynthesis‐related genes (Data Set S3), reflecting the distinct physiological conditions of phototrophic seedlings and heterotrophic roots. We reasoned that DE genes not shared between WT and mutants might point at processes disrupted upon RBP45 misexpression and thus potentially contribute to the altered root growth. However, GO term analysis of the corresponding gene sets showed no significant enrichment.

To validate the findings from the transcriptome‐wide DE analysis, we selected several candidate genes with root‐related functions. Determination of total transcript levels via reverse transcription‐quantitative polymerase chain reaction confirmed differential expression of those genes in at least one mutant line and tissue type each (Figure S10). The MADS box gene *AGAMOUS‐like 14* (*AGL14*) was previously found to modulate auxin transport and root development via regulation of *PIN* genes, resulting in reduced primary root length in *agl14* mutants (Garay‐Arroyo et al., 2013). In line with this function, reduced *AGL14* levels were found in several of our mutants, with the most pronounced downregulation seen in the *rbp45abc* mutant. Diminished expression of another growth‐promoting factor was detected in the case of *POPEYE (PYE)*, encoding a bHLH transcription factor regulating iron homeostasis genes under iron‐deficient conditions (Long et al., 2010). Moreover, we validated diminished transcript levels of the putative E3 ligase gene *BRUTUS* (*BTS*) that has been reported as a coregulator of PYE in the response to iron deficiency (Long et al., 2010). Finally, we validated for *OE‐C* seedlings the upregulation of the transcription factor gene *ERF56*, which is known to be induced upon nitrogen supply (Varala et al., 2018; Yao et al., 2025). Interestingly, an opposite change was found in the corresponding root sample, suggesting tissue‐specific regulation that might reflect altered root/shoot partitioning of nitrogen in the respective *rbp45* mutants. Taken together, we validated that upon RBP45 misexpression several genes related to root development and nutrient homeostasis were deregulated, which might contribute to or be a consequence of the diminished root growth.

In addition to the DE analysis, we determined the numbers of differentially alternatively spliced (DAS) genes by comparing the mutant lines to WT, resulting in the seedling and root datasets, respectively, in 51 and 45 affected genes (Figure 7c; Data Set S2). Considering the AS changes upon RBP45 OE, *RBP45B* resulted in most changes among the three corresponding mutants with 14 DAS genes in whole seedlings (Figure 7d). No DAS genes were detected for the *RBP45A* OE seedlings, whereas the *RBP45A* gene was the only DAS target in the *OE‐C* line, further supporting the cross‐regulatory mechanism (Figure 7d). For seedlings of the knockout mutants *rbp45bc* and *rbp45abc*, respectively, 8 and 40 DAS genes were detected, with 6 DAS genes shared between the two mutants (Figure 7d). A similar extent of AS changes was found in the root dataset, with 13 and 39 DAS genes, respectively, in the double and triple mutant and 7 overlapping genes (Figure S11a). Upon *RBP45C* OE, two DAS genes were found in the roots, of which one each overlapped with the dataset for the *rbp45bc* and the *rbp45abc* mutant. To further characterize the AS changes, we assigned differential transcript usage (DTU) transcripts to the identified DAS genes. In the cases where expression levels of two transcripts from the same gene were significantly and reciprocally altered in at least one type of misexpression line compared to the WT, AS event types and their genomic locations were determined (Data Set S2). This annotation was performed using the *A. thaliana* TAIR 10 genome and the AtRTD2‐QUASI reference transcriptome. Considering the AS event positions relative to the open reading frame, approximately half of the significantly altered events were located in the 5′ and 3′ UTR (Figure 7e; Figure S11b,c). This enrichment compared to the reference data set suggests that RBP45‐mediated AS to a major extent affects non‐coding regions.

**Figure 8:**
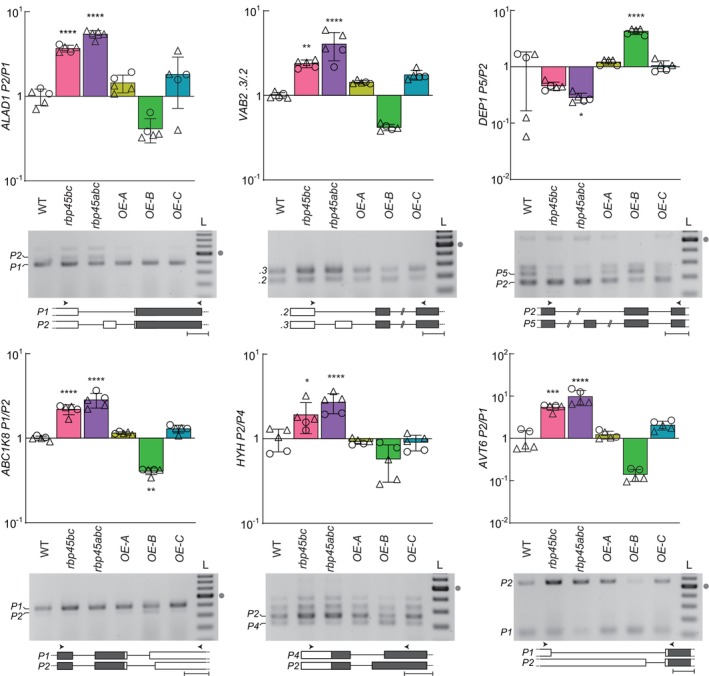
Reciprocal alternative splicing (AS) shifts upon *RBP45B* knockout and overexpression confirm its function in splicing regulation. AS ratios were analysed from 10‐day‐old *Arabidopsis thaliana* seedlings for *ALAD1* (*AT1G69740*), *VAB2* (*AT4G38510*), *DEP1* (*AT5G53850*), *ABC1K8* (*AT5G64940*), *HYH* (*AT3G17609*) and *AVT6* (*AT3G30390*). For each AS event, bar chart based on Bioanalyzer quantification (top) and representative gel picture (middle) of RT‐PCR co‐amplification products, and the corresponding gene models (bottom) are displayed. Mean value (bars), standard deviation (error bars) and individual data points (circles: based on samples used for RNA sequencing; triangles: additional replicates) are depicted each; mean AS ratio of wild‐type (WT) was set to 1. Asterisks indicate significant change compared to WT (one‐way anova followed by Dunnett's multiple comparisons test, *P < 0.05, **P < 0.01, ***P < 0.001, ****P < 0.0001). Size ladder (L) for gels consisted of DNAs in 100 bp increments with the strongest band corresponding to 500 bp (marked with grey dot). In the gene models, exons, introns, CDS and untranslated regions (UTRs) are depicted by boxes, lines, black and white shading, respectively; double dashes indicate cropped regions; arrowheads show primer binding sites and scale bar is individually adjusted to 100 bp for each model.

Seven candidate DAS events were validated using RT‐PCR (Figure 8; Figure S12). Out of those, three events were significantly changed in both RNA‐seq datasets (*VAB2, HYH* and *AVT6*), three (*ALAD1*, *DEP1* and *ABC1K8*) were unique to whole seedlings and one (*XBAT35*) was only detected in the root dataset. According to the RNA‐seq data, only *DEP1* and *AVT6* exhibited significant reciprocal splicing changes in comparison of the two knockout lines and the *OE‐B* lines. In our validation experiments, all seven AS events were tested across all genotypes, and the significant changes from the RNA‐seq were independently confirmed (Figure 8; Figure S12). In the case of *rbp45bc* and *rbp45abc*, all candidate events were significantly and consistently altered both in the two types of mutants and tissues. With respect to the quantitative change, the effect was slightly but consistently more pronounced in the triple mutant, suggesting an additive effect of RBP45 paralog loss. Interestingly, an in general opposite AS shift was seen for *OE‐B*, while OE of the other two RBP45 paralogs did not have such an effect (Figure 8; Figure S12). This further supports a prominent function of *RBP45B* in AS regulation among the RBP45 family and is consistent with its central role in auto‐ and cross‐regulation. Regarding the potential physiological functions of RBP45‐dependent regulation, the AS change in *VAB2* encoding the vacuolar ATPase subunit B was particularly interesting given the essential role of this proton pump in processes such as nutrient uptake and development. Future studies need to address the mechanisms underlying RBP45‐dependent regulation of those candidate genes via AS and differential gene expression, and how this is linked to the physiological functions of this RBP family.

## DISCUSSION

### Crosstalk of RBP45 homologs via AS regulation

Here, we characterized the role of the strucRNA *45ABC* in AS regulation of *RBP45A*, *RBP45B* and *RBP45C* from *A. thaliana*. Our results revealed negative auto‐ and cross‐regulation of these three genes via AS, balancing the ratio of protein‐coding *cd* variants to non‐productive *nc* variants that are targeted by NMD (Sack et al., 2025). This regulatory mechanism is common among RBP genes and enables rapid and dynamic fine‐tuning of protein levels (Müller‐McNicoll et al., 2019; Reddy et al., 2013; Wachter et al., 2012). In line with autoregulation, constitutive OE of any RBP45 member led to reduced levels of the *cd* isoform for the corresponding endogenous gene (Figure 3). With respect to cross‐regulation, *RBP45B* seems to play a dominant role among the three homologs. Its OE resulted in an increase of the *nc* variants for all three RBP45 genes, whereas in lines overexpressing *RBP45A* or *RBP45C* both the *cd* and *nc* isoform levels from all three endogenous RBP45 genes were diminished (Figure 3b,c). Accordingly, formation of the non‐coding variants from these genes is to a major extent caused by *RBP45B*, which in turn is suppressed in the *RBP45A* and *RBP45C* OE lines. This regulatory hierarchy is further supported by the analysis of the knockout mutants (Figure 4). Knocking out only *RBP45B* reduced *nc* isoforms for all three genes, whereas total levels of *RBP45A* and *RBP45C* transcripts were elevated. In the *rbp45bc* double mutant, however, *RBP45B nc* levels were restored to a WT‐like level. In parallel, the AS pattern of *RBP45A* was altered in the double mutant, with an increase of *cd* and decrease of *nc* transcripts, pointing towards a release of negative cross‐regulation by *RBP45C*. As a consequence, *RBP45A* was de‐repressed in the double mutant and triggered enhanced production of *nc* transcripts from the *RBP45B* gene (Figure S13). Moreover, the *rbp45abc* triple mutant had strongly diminished amounts of *nc* isoforms for all three genes, suggesting that this regulatory circuit is probably confined to the three RBP45 genes.

Similar auto‐ and cross‐regulatory circuits have been identified in plants before, including studies of the hnRNP genes *GRP7* and *GRP8* (Schöning et al., 2007, 2008) and the three *PTB* genes (Rühl et al., 2012; Stauffer et al., 2010) from *A. thaliana*. AS‐based cross‐regulation was observed for *GRP7*/*GRP8* and *PTB1*/*PTB2*, whereas the more distantly related *PTB3* had no effect on the splicing outcome of its two paralogs. What is then the molecular mechanism underlying the hierarchy of the more complex regulatory circuit shown in this study for the RBP45 genes? And why does it involve a conserved structured mRNA motif as opposed to a simpler pre‐mRNA architecture containing single or several binding sites for the corresponding RBP, as has been demonstrated in other instances?

Evolutionary analyses indicate that *RBP45B* is the most ancient one of the three paralogs, while *RBP45C* and *RBP45A* emerged more recently and are more closely related (Lorković et al., 2000; Park et al., 2006). This relationship is also reflected by the different domain organization of the proteins. All three RBP45 proteins contain three RRMs; while RBP45A and RBP45C have in addition a glutamine‐rich N‐terminus, RBP45B is enriched in glutamine residues near the C‐terminus and exhibits a proline‐rich N‐terminus. These differences might be responsible for or contribute to the distinct roles of the three RBP45 genes in AS regulation. Moreover, varying total and spatial expression patterns might play a role, and we observed in WT plants that the transcript levels for *RBP45B* were highest among the three homologs (Data Set S2). A major role of *RBP45B* in AS regulation is not only supported by its function in the auto‐ and cross‐regulatory circuit, but also by our transcriptome‐wide analyses. *RBP45B* OE caused the highest number of significant AS changes (Figure 7) as well as reciprocal AS shifts compared to the double and triple knockout mutants (Figure 8). Given that even for the *RBP45B* OE line and the triple mutant *rbp45abc* relatively few global AS changes were detected, redundancy with other RBPs and/or additional functions of these RBP45 genes besides AS regulation can be assumed. Moreover, our observation of specificity among the RBP45 genes indicates unequal genetic redundancy, which has been previously discussed to be a more common evolutionary process of functional gene diversification in *A. thaliana* (Briggs et al., 2006).

### Critical and interconnected roles of *45ABC* sequence and structure in balancing RBP45 expression

The exclusive presence of the strucRNA *45ABC* in the three RBP45 genes and the associated cross‐regulatory network suggested that their expression is coordinated in an RNA structure‐dependent manner. RNA folds can affect RBP interactions by various means, for example through exposing or occluding binding sites, while RBP binding in turn can remodel the RNA structure. Recent transcriptome‐wide structure‐profiling studies in plants further highlighted the critical interplay between RNA sequence and structural features. For example, Gosai et al. (2015) observed that RBP binding sites in nuclear mRNAs are generally less structured, and Liu et al. (2021) reported that the 5′ splice site and branch point need to be single‐stranded for efficient splicing. In plants, few examples exist where the impact of RNA structure and its dynamical behaviour on AS or other steps of gene expression has been functionally characterized. One such case is the TPP riboswitch mechanism that functions via occlusion of the alternative 5′ splice site by the interaction with an aptamer region in the absence of ligand binding (Wachter et al., 2007). AS regulation via changes in splice site availability was also reported for the heat shock factor *HsfA2* gene from tomato, involving temperature‐dependent structural changes at a 3′ splice site (Broft et al., 2022). An alternative mechanism has been proposed in AS control of the *TFIIIA* gene via the strucRNA *P5SM* (Hammond et al., 2009), where L5 binding may prevent binding of a splicing regulatory protein independent of an RNA structural change.

**Figure 9:**
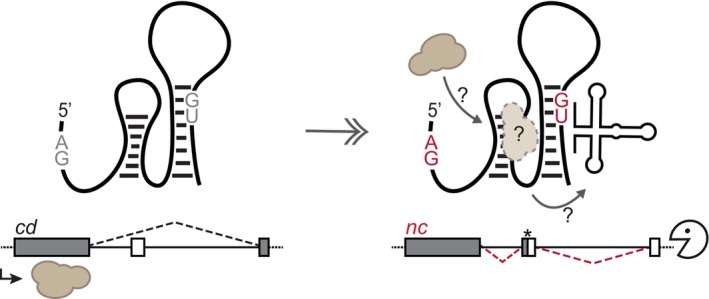
Proposed model of *45ABC*‐mediated alternative splicing (AS) regulation. The strucRNA *45ABC* consists of two stem loops, with the second one encompassing the alternative 5′ splice site used for cassette exon (CE) inclusion. When RBP45 protein (brown cloud) is absent (left), the CE is removed resulting in the *cd* variant that is translated into RBP45 protein. In the presence of RBP45, it might bind to stem I and facilitate U1 snRNP recruitment to the alternative 5′ splice site in stem II (right), thereby promoting its usage. The CE‐containing *nc* variant contains a premature termination codon (asterisk), triggering nonsense‐mediated decay (NMD) turnover as part of the negative feedback regulatory loop. Grey and white boxes in the transcript models correspond to coding and non‐coding regions.

Using a mutational approach, we showed in this work that opening the structure of *45ABC* resulted in increased usage of the alternative splice sites (Figure 5). The most pronounced shift towards the *nc* isoform including the CE was observed upon disruption of stem I. Compensatory mutations that restored base‐pairing also rescued the splicing pattern to a WT‐like AS ratio, underscoring the structural requirement for proper splicing regulation by this element. The important role of stem I in AS regulation combined with the localization of the alternative 5′ splice site and the respective U1 snRNP‐binding motif (Sheth et al., 2006) within stem II suggest that the assembly of the two hairpins is required for proper splice site selection. Sequence and structure conservation of *45ABC* may therefore be explained by its several roles; sequence motifs for recruitment of U1 components and potentially RBP45, its structural constraints and potential crosstalk between the elements. Notably, all three RBP45 proteins have been shown to interact with the U1 snRNP component U1‐C (Huang et al., 2022). Their human orthologue TIA‐1 is described to bind downstream of the 5′ splice site and to recruit U1 snRNP through a direct interaction with U1‐C, thereby influencing splice site selection (Förch et al., 2002). In the case of the RBP45 proteins, their proposed binding to stem I of *45ABC* might facilitate U1 recruitment to the alternative 5′ splice site embedded in stem II and thereby promote its usage. Accordingly, this strucRNA may act as a sensor for RBP45 protein levels that allows balancing RBP45 expression in an AS‐dependent manner (Figure 9). Closely related proteins, namely RBP45D and RBP47 (Chang et al., 2022), could potentially also bind *45ABC* and affect the splicing outcome. RBP45D has been reported to be involved in alternative splice site selection via interaction with U1 components (Chang et al., 2022), whereas the RBP47 proteins have been described as regulators of stress granule dynamics and therefore may primarily act in other aspects of RNA metabolism (Kosmacz et al., 2019). Functional redundancy between RBP45 and possibly also RBP47 proteins in U1 recruitment to 5′ splice sites could also explain why even in the triple mutant *rbp45abc* only relatively few AS changes were detected in our transcriptome‐wide analysis. Besides being part of the spliceosome, U1 snRNP also performs splicing‐independent functions. In a process termed telescripting, U1 binding blocks recognition of cryptic polyadenylation signals and thereby prevents premature cleavage and polyadenylation (Berg et al., 2012; Kaida et al., 2010). This additional U1 snRNP function has recently also been demonstrated for *A. thaliana* (Mangilet et al., 2024) and future experiments should address a possible involvement of RBP45 proteins.

We also examined if the conserved purine‐rich motif (RGRG) within stem I might serve as a binding site for the three RBP45 proteins and is important for the regulation. In line with this hypothesis, transient reporter assays revealed that mutating the five consecutive guanines to adenines caused an almost complete loss of the *nc* isoform under control conditions (Figure 5). Upon co‐expression of any *RBP45* CDS, a splicing response was still detectable, but it was markedly attenuated compared to the WT construct. This residual responsiveness may be explained by RBP45 binding to the introduced U stretch, consistent with previous findings that recombinantly expressed RBP45 from *N. plumbaginifolia* (Lorković et al., 2000) and RBP45D from *A. thaliana* (Huang et al., 2022) interacted with U‐rich RNAs *in vitro*. Alternatively, or additionally, it might reflect non‐specific interactions caused by high levels of RBP45 expression in the transient expression system. Accordingly, stable *A. thaliana* lines carrying the BM2 splicing reporter showed exclusive splicing towards the *cd* variant. With regard to the proposed binding preference of the RBP45 proteins, the U‐rich loop of hairpin I (Sack et al., 2025) may also contribute to RBP45 binding. Furthermore, it is well known that RBPs differ not only in their sequence preferences but also with respect to the structural requirements at the binding site and its context (Dominguez et al., 2018; Foley et al., 2017; Gosai et al., 2015). Accordingly, conservation of the stem I structure of *45ABC* might be a consequence of the RBP45 proteins' binding preferences. Taken together, both sequence and structural features of *45ABC* contribute to this RBP45‐responsive regulatory mechanism, providing also an explanation for the relatively low level of covariation of this motif (Sack et al., 2025).

Our findings raise the question whether related mechanisms of strucRNA‐dependent AS are more common. Interestingly, Sack et al. (2025) also identified conserved strucRNAs within the pre‐mRNAs of *GRP7*, *GRP8* and *PTB2*. The *GRP7&8* strucRNA consists of a hairpin including an alternative 5′ splice site in the predicted stem (Sack et al., 2025). Usage of this splice site leads to NMD‐sensitive isoforms as part of negative feedback regulation (Schöning et al., 2008), and both GRPs have been shown to bind downstream of this motif (Leder et al., 2014; Staiger et al., 2003). Accordingly, the corresponding strucRNA may suppress usage of this splice site, while GRP binding could promote it (Sack et al., 2025). Similarly, the *PTB2* motif is a single hairpin that includes the 5′ splice site and a polypyrimidine stretch that are used for AS‐mediated negative auto‐ and cross‐regulation of *PTB2* expression (Burgardt et al., 2024; Sack et al., 2025; Stauffer et al., 2010). So far, experimental evidence for the functional relevance of those novel strucRNAs is lacking. However, their discovery suggests that such RNA folds might be more common in AS regulation. Reasons for their sequence and structural conservation can include the presence of binding sites for splicing factors and regulators, specific requirements for structuredness, and regulatory interactions between individual elements as proposed here for *45ABC*. Moreover, shorter motifs such as single hairpins are more difficult to detect by bioinformatics approaches and thus might be more common than reflected by our current knowledge.

### Physiological roles and functions of RBP45 proteins

Our transcriptome‐wide analyses of *rbp45* knockout and OE lines revealed only limited changes in global gene expression and AS. Interestingly, most observed AS changes occurred in UTRs (Figure 7; Figure S11). This enrichment could point at additional functions of RBP45 association with its target RNAs that occur downstream of AS control, such as the established role of UTR‐binding proteins in processes such as mRNA translation, localization and stability (Hardy & Balcerowicz, 2024). Consistent with such an idea, RBP45 proteins localize to both the nucleus and cytoplasm (Huang et al., 2022; Lorković et al., 2000) and interact with mRNA decay components such as UPF1 (Chicois et al., 2018; Sulkowska et al., 2020). Protein–protein interaction studies further support a role of RBP45 proteins in regulating the mRNA fate, for example RBP45B was shown to interact with the cap‐binding protein CBP20 and the poly(A)‐binding protein PAB8 (Muthuramalingam et al., 2016). Furthermore, RBP45A, RBP45B and RBP45C were reported to be associated with the UTRs of the *GRP7* mRNA (Reichel et al., 2024). These interactions and the concomitant regulatory potential might be particularly important under stress. Accordingly, expression of *RBP45A* and *RBP45B* has been reported to be induced upon ozone exposure (Peal et al., 2011).

Critical functions of the RBP45 genes in plant development are supported by the mutants' phenotypes. With an increasing number of knocked out RBP45 genes, we observed a progressively reduced primary root length (Figure 6a). This observation also suggests functional redundancy among the three paralogs. Interestingly, OE of *RBP45C* similarly disturbed root growth. Inspecting the transcript levels in this OE line from the RNA‐seq data revealed a massive overaccumulation of *RBP45C* specifically in the root, pointing at tissue‐specific regulation (Data Set S2). This overaccumulation may have caused suppression of the other two RBP45 paralogs and therefore resulted in a similar phenotype as for the knockout lines. Several of the RBP45 target genes validated by us contribute to root development and function, providing possible mechanistic links between the observed alterations in gene expression and root development. Further research is needed to validate additional potential target genes and to provide direct evidence for their functional relevance. Interestingly, a previous study by Foley et al. (2017) identified an interaction of RBP45 proteins with a TG‐rich motif in the 3′ UTRs of root hair‐specific transcripts, indicating a role of RBP45 function in root cell fate decisions. Beyond changes in root length, *rbp45* mutants showed altered flowering time. OE of *RBP45A* caused a mild delay, while *RBP45B* and *RBP45C* OE led to earlier and later flowering, respectively (Figure 6b). Delayed flowering upon loss of *RBP45B* is consistent with previous observations (Muthuramalingam et al., 2016). Interestingly, this phenotype was absent in the higher‐order mutants, suggesting a potential antagonism or compensatory functions among *RBP45* homologs in this process.

In conclusion, our work has identified novel physiological functions of the RBP45 genes in plant development. Furthermore, we have characterized an intricate auto‐ and cross‐regulatory circuit that balances the expression of the three RBP45 genes via the strucRNA *45ABC*. The conserved sequence and structural features of this element point towards an AS‐regulatory mechanism that may involve RBP45‐mediated recruitment of U1 snRNP to an alternative splice site, resulting in negative feedback control of RBP45 expression.

## MATERIALS AND METHODS

### Plant cultivation and transformation

*Arabidopsis thaliana* ecotype Columbia‐0 plants were either grown on soil or in sterile culture on agar plates. Therefore, seeds were surface‐sterilized using 3.75% NaClO and 0.01% Triton X‐100 and stratified at 4°C for 2–4 days. If not specified otherwise, they were germinated on ½ Murashige and Skoog medium including vitamins (Duchefa, Haarlem, Netherlands) and containing 2% sucrose and 0.8% phyto agar. Segregating mutant lines were grown on plates containing additionally 25 μg ml^−1^ kanamycin. For experiments in which root lengths were measured, 1.2% phyto agar was used and plates were placed not in horizontal but vertical orientation. The standard settings of the climate chambers were 16 h white light (~100 μE)/22°C and 8 h darkness/20°C at 60% relative humidity. *Nicotiana benthamiana* was grown on soil in a climate chamber (16 h white light (~120 μE)/24°C, 8 h dark/22°C at 60% relative humidity).

Stable transformation of *A. thaliana* was achieved by the floral dip method described by Clough and Bent (1998). Selection of primary transformants was done on half‐strength Murashige and Skoog medium (including vitamins) containing 0.8% plant agar, 25 μg ml^−1^ kanamycin and 200 μg ml^−1^ cefotaxime. Resistant plants were transferred to soil and, upon further growth, subjected to PCR‐based genotyping. Selection of CRISPR lines was carried out via the fluorescence‐accumulating seed technology (FAST; Shimada et al., 2010) under a Leica (Wetzlar, Germany) M205FCA fluorescence stereomicroscope (excitation: 541–551 nm, emission: 565–605 nm) and positive seeds directly germinated on soil. Cas9‐free plants in later generations were confirmed by the absence of fluorescence.

*Nicotiana benthamiana* leaves were transformed via Agrobacteria‐mediated leaf infiltration as described by Wachter et al. (2007) with a normalization construct based on mOrange2 (Shaner et al., 2008). Leaf material was harvested 2 and 3 days after infiltration, respectively, for analysis on RNA and protein levels.

### Genotyping of plant mutants

The genetic status of the generated mutants was confirmed by isolating genomic DNA followed by genotyping PCR. To 100 mg ground plant tissue, 500 μl extraction buffer (200 mM Tris–HCl pH 9, 400 mM LiCl, 25 mM EDTA, 1% SDS) was added and vortexed thoroughly at room temperature. After 5 min centrifugation at 15 000 ***g***, the supernatant was mixed with the same volume of isopropanol, incubated for 5 min, and then centrifuged for 10 min at 15 000 ***g*** to precipitate the gDNA. The pellet was washed with 70% ethanol, dried at 37°C and resuspended in ½ TE buffer (5 mM Tris–HCl pH 8, 0.5 mM EDTA). Genotyping PCR was performed with homemade Taq polymerase according to standard procedures with primers listed in Data Set S4. For CRISPR lines, a proof‐reading polymerase was used for PCR amplification, followed by purification of PCR fragments (GeneJET PCR Purification Kit, Thermo Fisher Scientific, Waltham, Massachusetts, USA) and Sanger sequencing of mutated sites.

### Plant phenotyping

Seeds were sorted using Boxeed 2.1 (Labdeers, Boskovice, Czech republic) with the following criteria: pixel count range 200–2000, SSE 0–95 and LS ratio 0–25. Measurements and pictures of matching seeds were taken, and size distribution was analysed.

For root length measurements, seeds were also stratified for 96 h in darkness at 4°C before plating them on square ½ MS plates singly in one row. The plates were placed vertically into racks and kept under the aforementioned conditions for 7 days. The plates were scanned, and primary root length was measured using ImageJ software (Schindelin et al., 2012).

For the determination of flowering time and monitoring development, seeds were stratified for 96 h in darkness at 4°C and then cultivated on soil under the conditions mentioned above. Flowering time was defined as the time span at which the inflorescence reached a length of 1 cm. Rosette diameters were measured at different time points after sowing and the number of rosette leaves was counted at the onset of flowering.

### Cloning procedures

The splicing reporters for *RBP45A* (*AT5G54900*), *RBP45B* (*AT1G11650*) and *RBP45C* (*AT4G27000*) were constructed by PCR amplification of the corresponding sequences from *A. thaliana* genomic DNA using the oligonucleotides indicated in Data Set S4 and sub‐cloning into pGEM‐T (Promega, Madison, Wisconsin, USA, www.promega.de). All final reporter constructs were cloned in the pBinAR vector (Höfgen & Willmitzer, 1990) using the previously published *TFIIIA* reporter (Hammond et al., 2009) by replacing the target gene region via *Kpn*I/*Xba*I restriction digest. Motif mutations were introduced via PCR mutagenesis in pGEM‐T using oligonucleotides as indicated in Data Set S4. The CDS constructs used for leaf infiltration assays and generating the stable constitutive OE lines were also cloned in the pBinAR vector. Upon PCR amplification from cDNA, the fragments were first subcloned into pGEM‐T and subsequently ligated into pBinAR via *Kpn*I/*Xba*I. All final constructs were confirmed by sequencing of the inserts. The CRISPR‐Cas9 constructs for generation of *rbp45* knockout plants were created using the GoldenGate cloning system reported by Stuttmann et al. (2021). The sgRNAs indicated in Data Set S4 were designed using CHOPCHOP (Labun et al., 2019) and cloned into the pDGE652 or pDGE347 vector under control of the *A. thaliana U6‐26* promoter fragment as described by Stuttmann et al. (2021). The vector additionally features an intron‐containing *Cas9* under control of the *RPS5A* promoter.

### RNA isolation & analysis of splicing patterns

RNA was isolated from ~100 mg ground plant tissue using EURx Universal RNA Purification Kit (Roboklon, Berlin, Germany) with an on‐column DNase treatment (RNase free DNaseI, New England Biolabs, Ipswich, MA, USA) as specified in the manufacturer's protocol. The RNA concentration was determined using a spectrophotometer (DeNovix DS11 FX+, Wilmington, Delaware, USA). Reverse transcription was carried out with SuperScript II (Invitrogen, Thermo Fisher Scientific) using a dT_20_ primer with a 5 min incubation step at 65°C prior to adding the reaction buffer and enzyme. The cDNA obtained was subjected to analysis of splicing patterns via co‐amplification PCR or quantitative PCR (qPCR).

Co‐amplification PCR was performed with primers (Data Set S4) encompassing the alternative spliced regions and a homemade Taq polymerase. The resulting DNA products were separated on horizontal agarose gels or via native PAGE. Either a 1 kb plus (New England Biolabs) or a 100 bp plus GeneRuler ladder (Thermo Fisher Scientific) was used to estimate the molecular weight. The bands were stained after electrophoresis in ethidium bromide solution at 0.5 μg ml^−1^. The bands were visualized with UV light and documented with the Quantum‐CX5 Edge (Vilber). Quantification of co‐amplification PCR products was performed with a Bioanalyzer 2100 (Agilent, Santa Clara, CA, USA, www.agilent.com) using the DNA 1000 chip according to manufacturer's protocol.

qPCR was performed using the Bio‐Rad CFX384 Real‐Time PCR system (Bio‐Rad, Hercules, California, USA, www.biorad.com) and the MESA‐BLUE 2x qPCR MasterMix Plus for SYBR® Assay (Eurogentec, Ougrée, Belgium, www.eurogentec.com). Primers are listed in Data Set S4 and primer efficiencies were determined with serial dilutions of template. All reactions were done in triplicates, and a melting curve analysis was included. Analysis of the data was done with the use of the relative standard curve method. Transcript level of *PP2A* (*AT1G13320*) served as reference.

### Whole transcriptome sequencing

150 ng total RNA isolated as described before and derived from two biological replicates of 10‐day‐old *A. thaliana* WT, *rbp45abc, rbp45bc* and *RBP45A*/*RBP45B/RBP45C* OE seedlings was utilized. For root‐specific analysis, RNA was used from clipped roots of 7‐day‐old seedlings with three biological replicates each for WT, *rbp45bc*, *rbp45abc* and the *RBP45C* OE line. The concentration and quality of the RNA was assessed based on the RNA integrity number (RIN) measured with an Agilent Bioanalyzer 2100 using the RNA 6000 Nano protocol. Eurofins Genomics (Ebersberg, Germany) performed poly‐A‐selection and generation and sequencing of strand‐specific cDNA libraries on a NovaSeq 6000 S4 system, generating a minimum of 25 million read pairs (2 × 150 nt) per sample. BBDuk (Bushnell B., www.sourceforge.net/projects/bbmap) was used for trimming the reads, which were then mapped to the reference transcriptome AtRTD2‐QUASI (Zhang et al., 2017) using Salmon (Patro et al., 2017). Data were analysed using the 3D RNA‐seq App (Guo et al., 2021). Further details are given in Supporting Information—Methods.

To determine relative positions of AS events within transcripts, first the longest possible ORF from each transcript in AtRTD2‐QUASI was determined. For each gene, the transcript with the longest ORF was set as reference to define coordinates of translation start and end sites. Based on these coordinates, AS events were localized in all transcript isoforms per gene. Only the first event from the 5′ end was considered. The same events in multiple isoforms were counted only once. Further details are provided in Supporting Information—Methods and corresponding Python scripts are available at https://github.com/Christoph‐cloud‐ux/locate_AS.

Venn diagram displays were created with DeepVenn (Hulsen, 2022). Gene ontology term analysis was performed using ShinyGO 0.85 (Ge et al., 2020) with an FDR cut‐off of 1 × 10^−5^ using the KEGG pathway database (Kanehisa et al., 2021).

### Protein extraction and fluorescence assay

Total protein was isolated from ~100 mg ground tissue of infiltrated *N. benthamiana* leaves. After adding 300 μl buffer (50 mM Tris pH 7.5, 150 mM NaCl, 0.1% Tween, 0.1% ß‐mercaptoethanol), the samples were vortexed and centrifuged at 4°C for 15 min at 15 000 ***g***. Supernatant was transferred to a new tube and then 100 μl of each sample pipetted into a 96 well‐plate (black, flat bottom; Greiner, Kremsmünster, Austria) for fluorescence measurement. The fluorescence was detected using the TECAN (Männedorf, Switzerland) Infinite M1000, with excitation at 478–492 nm and emission at 515–525 nm for EGFP measurement. For fluorescence measurement of the reference mOrange2, the settings 525–530 nm for excitation and 590–610 nm for emission were used. Measurements were taken with flash frequency of 400 Hz and integration time of 20 μs. The measured values were read out in TECAN i‐control and transferred to Microsoft Excel (Microsoft Office 2016) for evaluation.

### Statistical analyses

Statistics were carried out with Prism GraphPad 9.4.0 (GraphPad; www.graphpad.com) or, in the case of seed surface data, using R Statistical Software (v4.1.2; R Core Team 2021). It was assumed that data follow a normal distribution. Results from statistical analysis are listed in Data Set S5.

## AUTHOR CONTRIBUTIONS

Conceptualization: AW, MR, ZW; Investigation: MR, RB, CE, MS; Writing: MR and AW; Supervision: AW and ZW; Funding acquisition: AW and ZW.

## CONFLICT OF INTEREST

None of the authors have a conflict of interest to disclose.

## Supporting information


**Figure S1.** Structural predictions of *45ABC* elements and RBP45 splicing patterns in different tissues.
**Figure S2.** Splicing patterns of RBP45 reporter constructs.
**Figure S3.** Models of RBP45 genes and sites of mutations in knockout lines.
**Figure S4.** *45ABC* mutations change splicing of *RBP45C* reporter in *N. benthamiana*.
**Figure S5.** Seed sizes and rosette growth in RBP45 misexpression lines and WT.
**Figure S6.** Principal component analyses of transcriptome datasets.
**Figure S7.** RBP45 misexpression causes relatively few changes in AS and gene expression.
**Figure S8.** A *Copia‐like retrotransposon* is downregulated in all *rbp45* misexpression lines.
**Figure S9.** Tissue‐specific gene expression in WT and *rbp45* misexpression lines.
**Figure S10.** Validation of differential gene expression in RBP45 knockout and overexpression lines.
**Figure S11.** DAS genes and AS event location in root samples upon RBP45 misexpression.
**Figure S12.** Validation of splicing patterns for candidate genes in RBP45 knockout and overexpression lines.
**Figure S13.** Model of crosstalk and hierarchy in RBP45‐mediated splicing regulation.

**Data Set S1.** Representatives of the *45ABC* motif.

**Data Set S2.** RNA‐seq data analysis.

**Data Set S3.** GO term analysis.

**Data Set S4.** List of oligonucleotides and constructs.

**Data Set S5.** Statistical analyses.

## Data Availability

RNA‐seq data have been deposited in the European Nucleotide Archive (ENA) repository (https://www.ebi.ac.uk/ena) under accession number PRJEB102517 and can be accessed via the following link: https://www.ebi.ac.uk/ena/browser/view/PRJEB102517.
