# Supplementary material for: The structured mRNA element 45ABC mediates auto‐ and cross‐regulation of RBP45 genes via alternative splicing
Source: Plant J. 2026 Aug 18;127(4):e71060. doi: 10.1111/tpj.71060 (PMC13485355; doi:10.1111/tpj.71060)
Supplement: Supplementary file 1 [file TPJ-127-0-s004.pdf]

Reinhardt et al.

SUPPLEMENTAL DATA

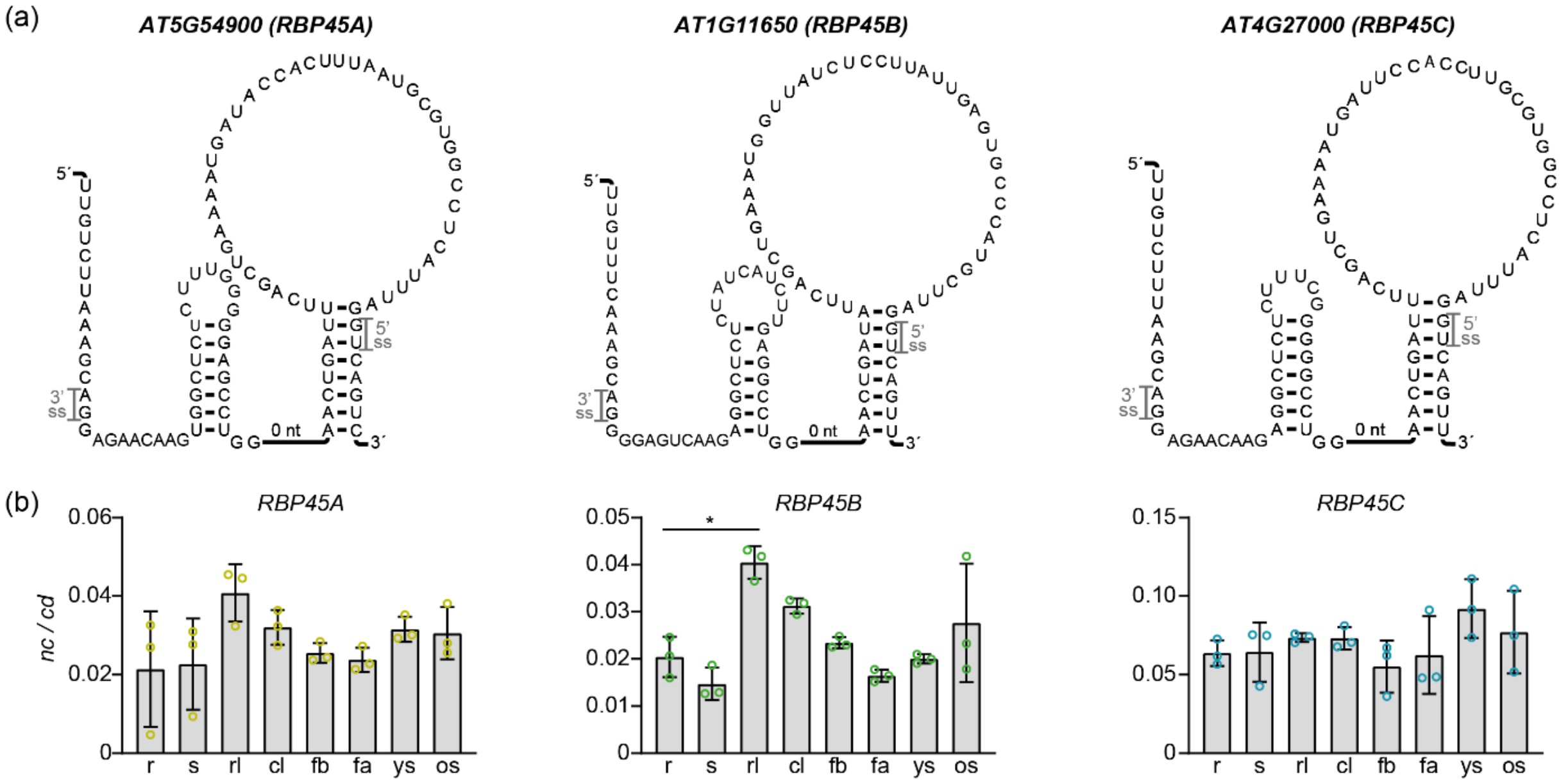

Figure S1: Structural predictions of *45ABC* elements and splicing patterns of corresponding RBP45 genes from *A. thaliana*.

(a) Sequences and structural predictions of the three *45ABC* representatives from *A. thaliana*. Alternative splice sites used for cassette exon inclusion are labelled.

(b) Bioanalyzer quantification of AS patterns for *RBP45A*, *RBP45B,* and *RBP45C* in different tissues from *A. thaliana* WT plants. r: seedling roots (7 d), s: total seedlings (12 d), rl: rosette leaves, cl: cauline leaves, fb: flower buds, fa: flower in anthesis, ys: young siliques, and os: old siliques. Circles represent biological replicates (n = 3); displayed are mean values of splicing variant ratios with standard deviation. Asterisk indicates significant difference between tissues; Kruskal-Wallis followed by Dunn’s test *$p < 0.05$.

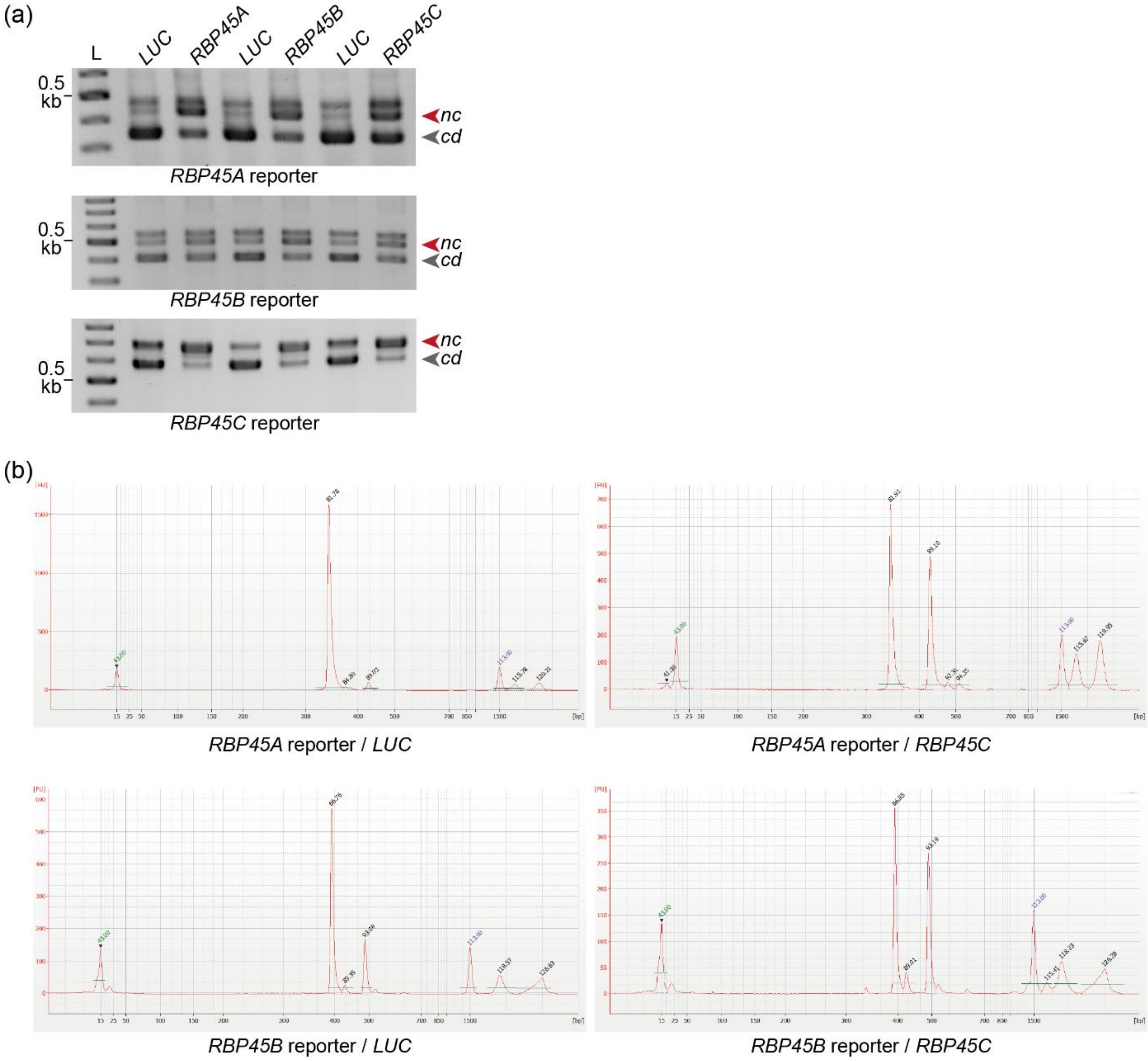

Figure S2: Splicing patterns of RBP45 reporter constructs.

(a) Agarose gel analysis of RT-PCR products derived from splicing reporters based on *RBP45A*, *RBP45B*, and *RBP45C* (top to bottom) upon transient expression in *N. benthamiana* leaves co-infiltrated with *LUC* or indicated CDS constructs. Splicing variants *cd* (*RBP45A*: 343 bp; *RBP45B*: 404 bp; *RBP45C*: 589 bp) and *nc* (*RBP45A*: 429 bp; *RBP45B*: 491 bp; *RBP45C*: 667 bp) are indicated. L: size marker, in 100 bp increments. The topmost band for the *RBP45A* and *RBP45B* reporters appeared only on Agarose gels and were undetectable from Bioanalyzer runs (b) and PAGE (Fig. 2b). Furthermore, sequencing RT-PCR products could identify only the indicated *cd* and *nc* splicing variants.

(b) Representative Bioanalyzer runs of RT-PCR products from splicing reporters as described in (a). Samples upon co-transformation with *LUC* (left) or *RBP45C* CDS (right) are shown for *RBP45A* reporter (upper) and *RBP45B* reporter (lower).

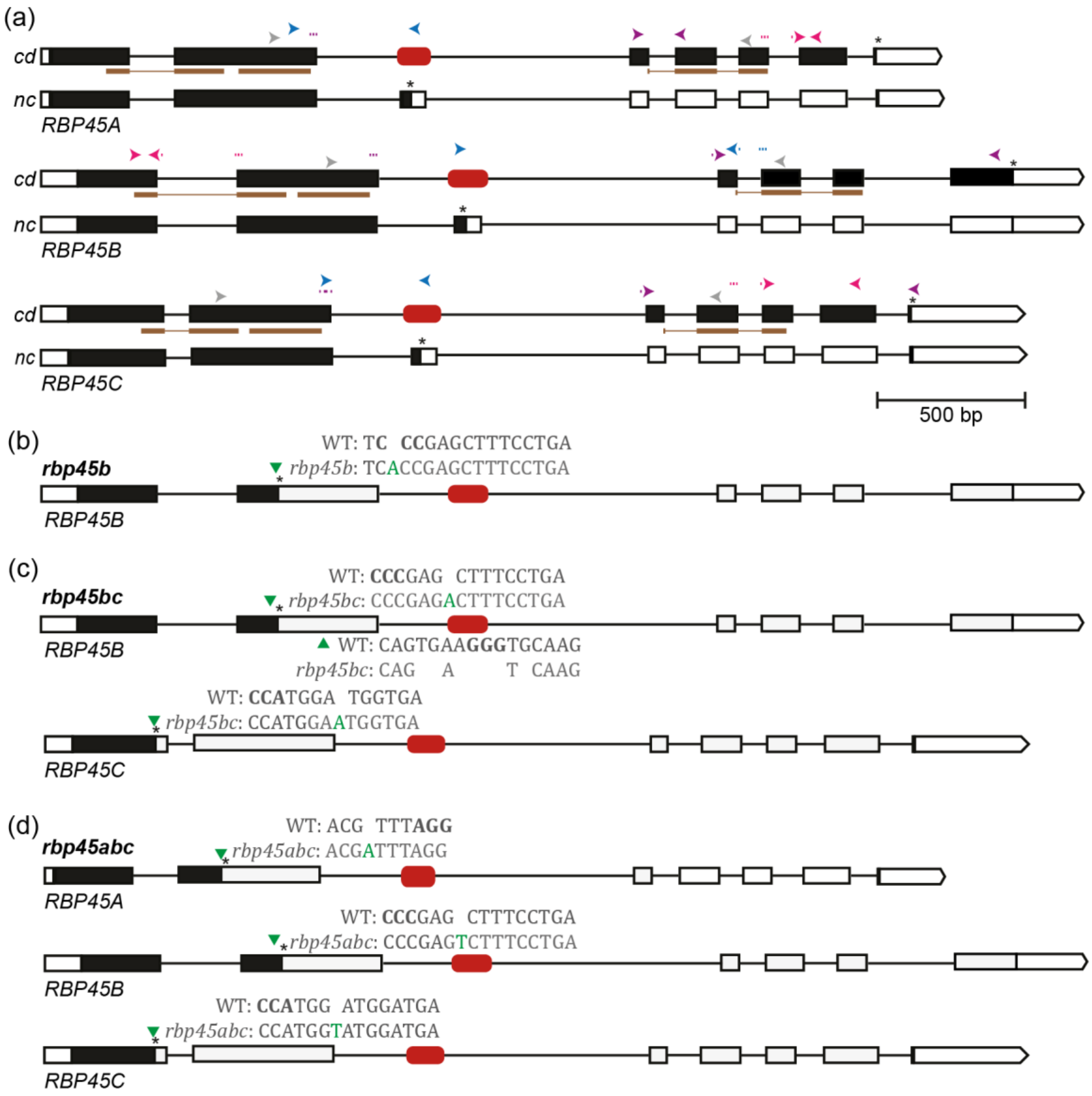

Figure S3: Models of RBP45 genes and sites of mutations in knockout lines.
Models of RBP45 genes from *A. thaliana* (a) and CRISPR-induced mutations in *rbp45b* (b), *rbp45bc* (c), and *rbp45abc* (d). Arrowheads show positions and directions of primers used for co-amplification PCR (grey) and qPCR (total levels (pink), non-coding (blue), and coding (purple)); dotted lines depict primers spanning exon-exon borders. Brown lines in (a) show regions coding for RRMs (according to UniProt.org). Exons, introns, CDS and UTRs are depicted by boxes, lines, black and white shading, respectively; *45ABC* motif is indicated by red rounded rectangle. Asterisks mark positions of regular or premature stop codons. Mutation sites are indicated as green triangles, and inserted sequences are shown in green letters; PAM sequence is shown in bold.

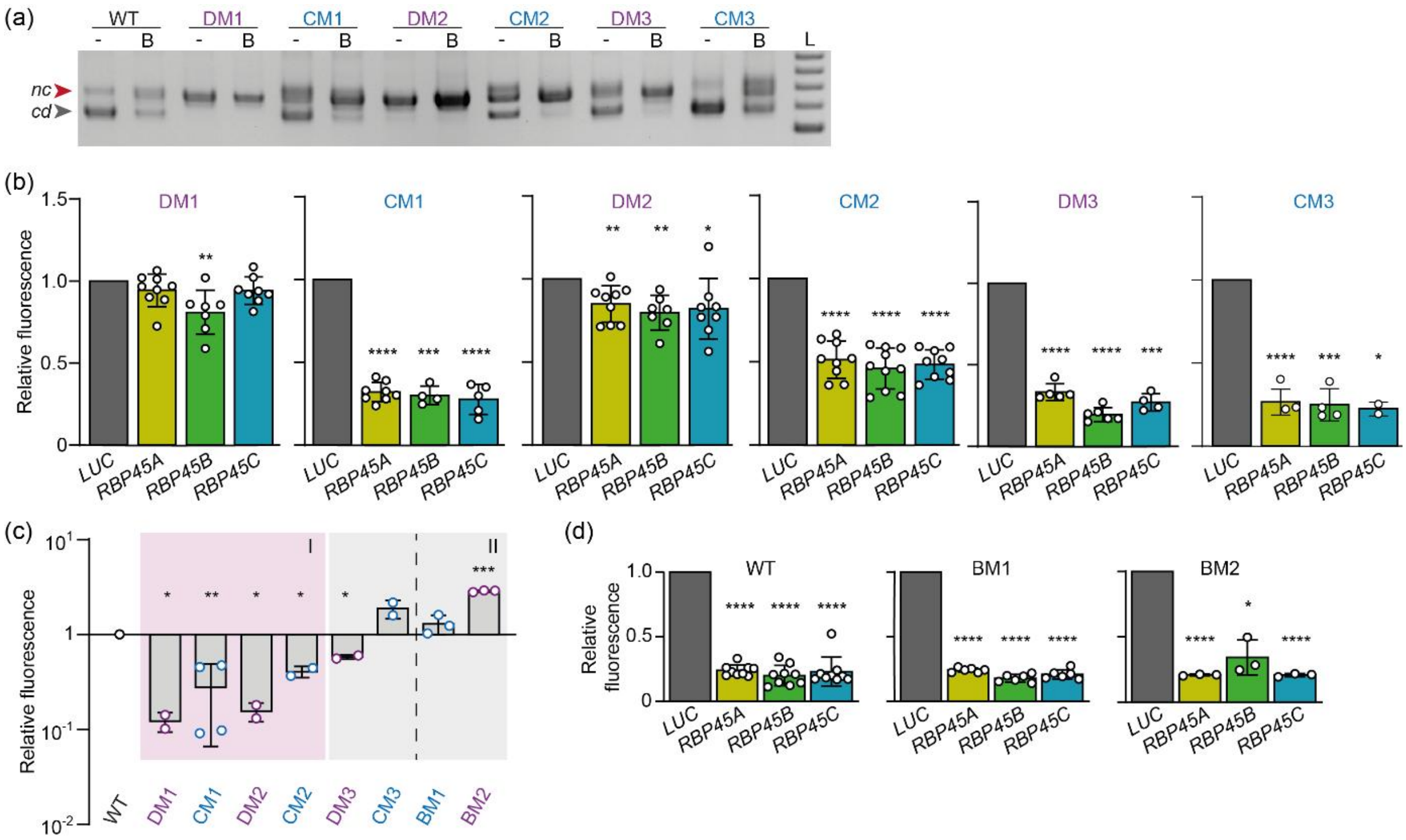

Figure S4: Mutations within *45ABC* change the splicing outcome of the *RBP45C* reporter upon transient expression in *N. benthamiana*.

(a) Agarose gel analysis of RT-PCR products of splicing variants from *RBP45C* WT and mutant reporters from *N. benthamiana* leaves co-infiltrated with *LUC* (-) or *RBP45B* (B) constructs. L: size marker, 100 bp increments from 500 – 900 bp.

(b – d) Quantification of reporter output based on GFP fluorescence from transiently transformed *N. benthamiana* leaves co-expressing the indicated splicing reporter with either *LUC* or the CDS constructs of *RBP45A*, *RBP45B*, and *RBP45C* (b, d), or with only LUC construct (c). Mean values and standard deviations are shown; open circles represent individual data points. Data normalization either separately for each reporter to the LUC sample (b, d) or to the mean of WT reporter (c). Asterisks indicate significant differences compared to the LUC control (b, d) or to the WT reporter (c) based on one sample t-test (*p < 0.05, **p < 0.01, ***p < 0.001, ****p < 0.0001).

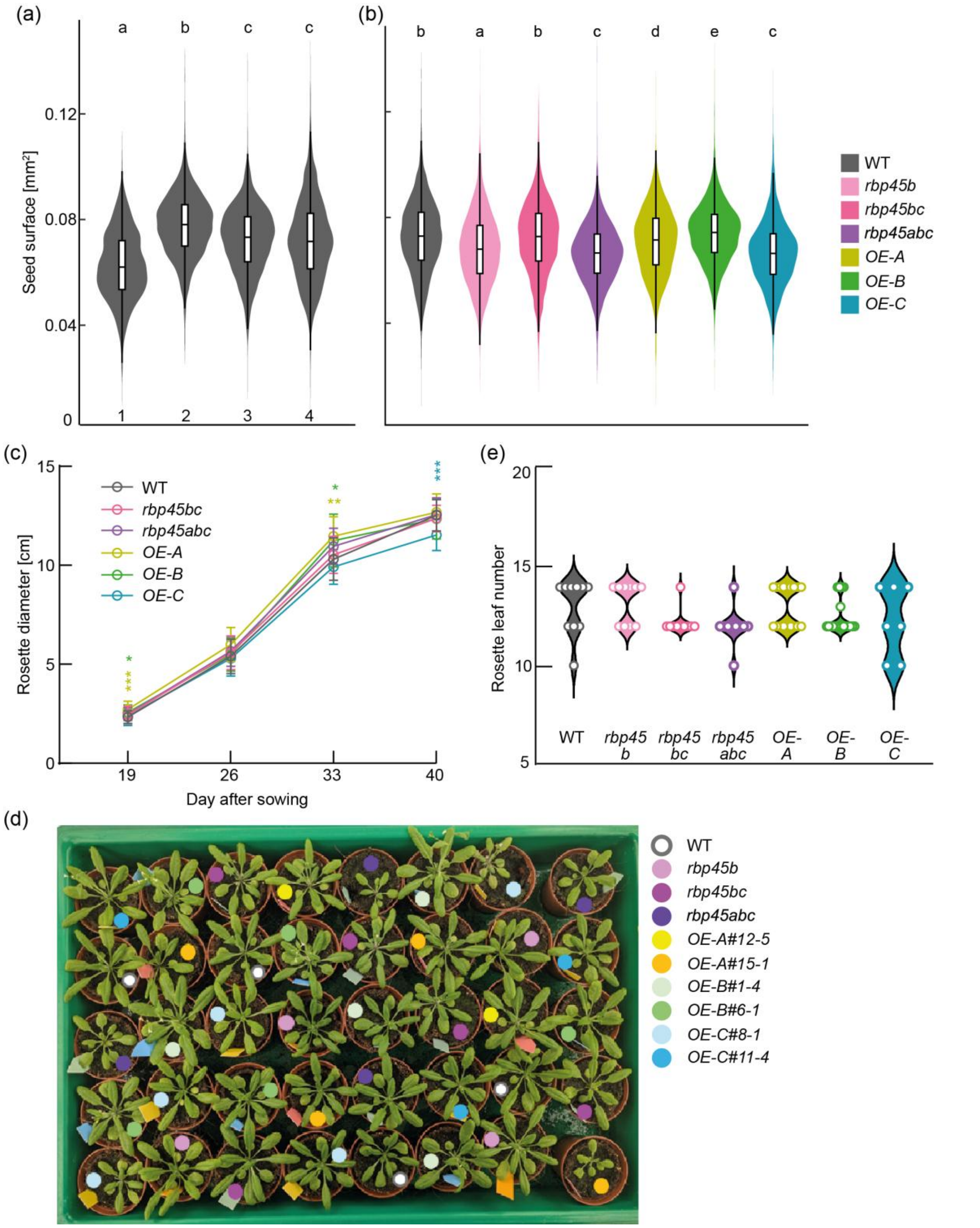

Figure S5: Seed size and rosette growth is similar in RBP45 misexpression lines and WT.
(a, b) Surface area of seeds from *A. thaliana* WT (4 batches from independently grown plants, a) and RBP45 misexpression lines (b). The WT seeds in (b) were a mixture from batches number 2 and 4 from (a). Number of seeds per sample was ≥ 3000 (WT) and ≥ 6000 (*RBP45* misexpression lines). Letters indicate statistically significant differences (ANOVA followed by Tukey test, $p < 0.05$). (c) Rosette diameter at indicated time points after sowing, depicted as mean values and standard deviations (n = 40 for *OE-A* and *OE-B*; n = 20 for the others). Asterisks indicate significant differences compared to WT based on Dunnett's multiple comparisons test (*p < 0.05, **p < 0.01, ***p < 0.001). (d) Representative photograph of 31-d-old plants. (e) Number of rosette leaves at onset of flowering as depicted in Figure 6b (n = 10 for WT, *rbp45b*, *rbp45bc* and *rbp45abc*; n = 18 for *OE-A*; n = 19 for *OE-B*; n = 7 for *OE-C*).

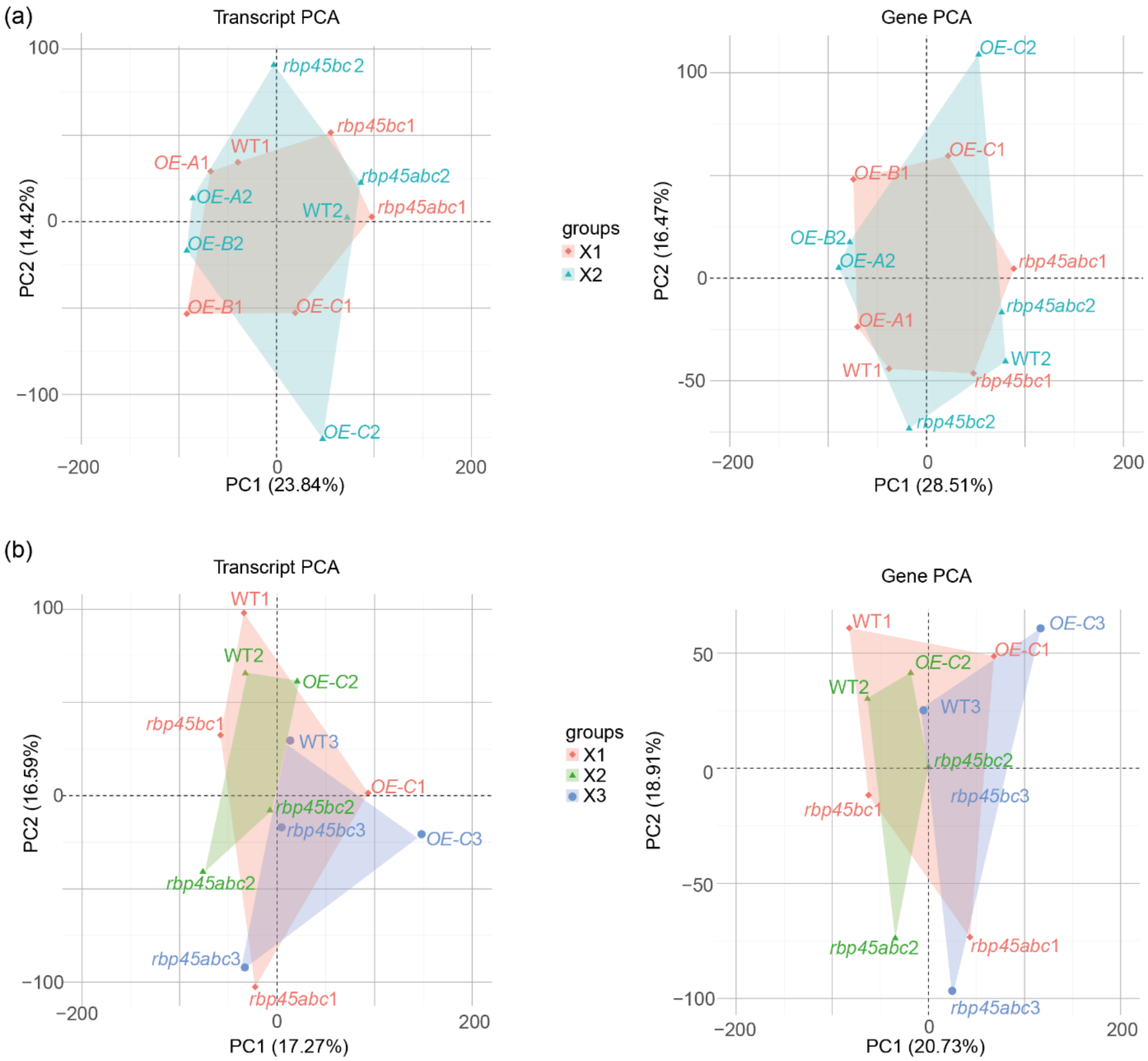

Figure S6: Principal component analyses of transcriptome datasets.
Transcript (left) and gene (right) PCA for datasets derived from 10-day-old whole seedlings (a) or roots from 7-day-old seedlings (b) for *A. thaliana* WT, *rbp45bc, rbp45abc*, and constitutive overexpression (OE) lines of *RBP45A, RBP45B*, and *RBP45C*. The suffix in the name refers to the replicate number. Note that the overexpression lines for *RBP45A* and *RBP45B* were only included in the whole seedling dataset in (a). The graphs were created as part of the data analysis in 3D RNA-seq.

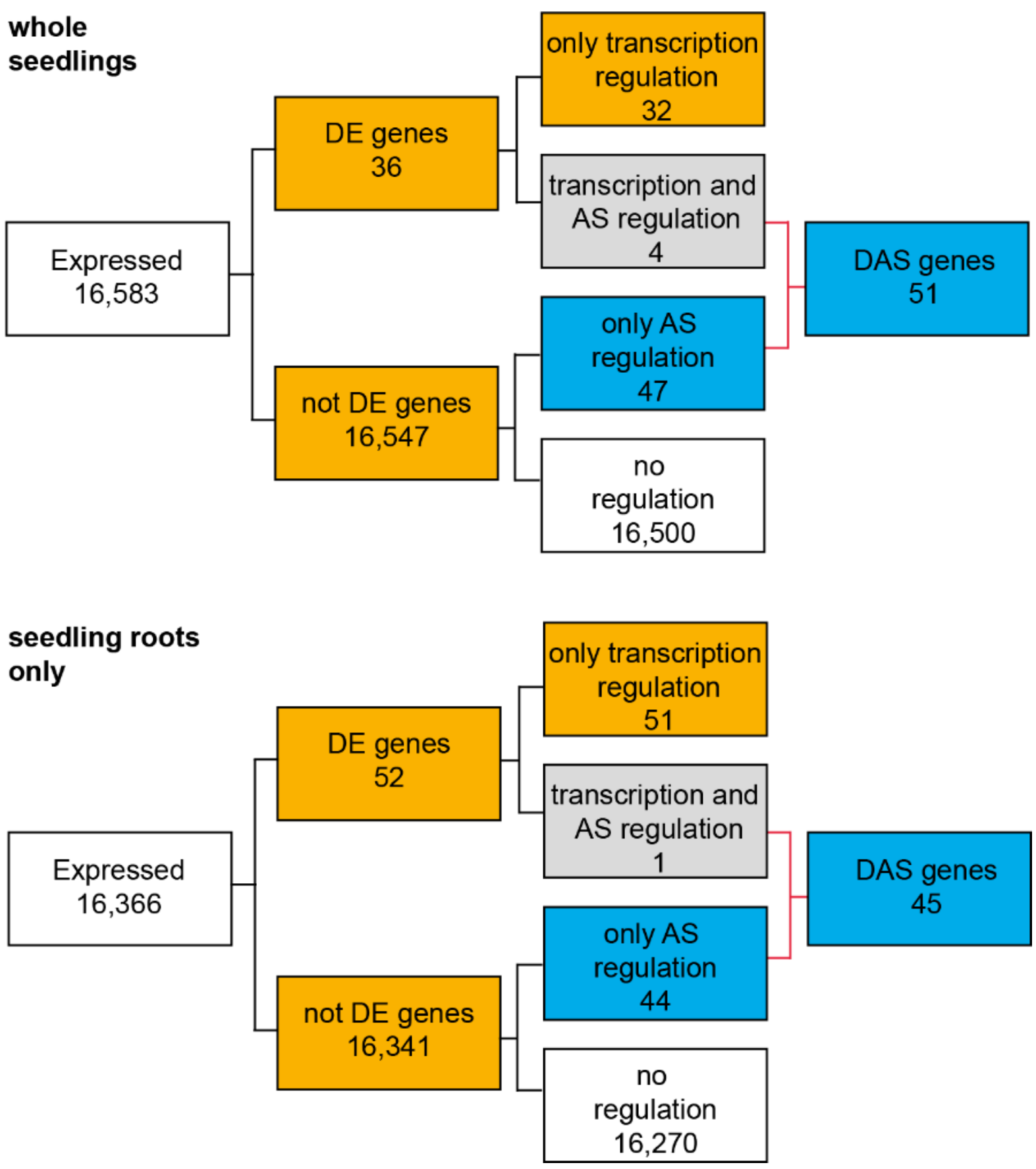

Figure S7: RBP45 misexpression causes relatively few changes in AS and gene expression. Numbers of expressed, differentially expressed (DE), and differentially alternatively spliced (DAS) genes in whole seedling (upper) and roots only (lower) samples. DE/DAS refers to changes in at least one RBP45 misexpression line relative to WT. The following mutant lines have been included; whole seedlings: *rbp45bc*, *rbp45abc*, *OE-A*, *OE-B* and *OE-C*; roots only: *rbp45bc*, *rbp45abc* and *OE-C* lines. The 3D RNA-seq analysis was performed with the following cut-offs for DE/DAS: p-value cut-off 0.01; log2 fold change cut-off 1; and delta PS cut-off 0.1.

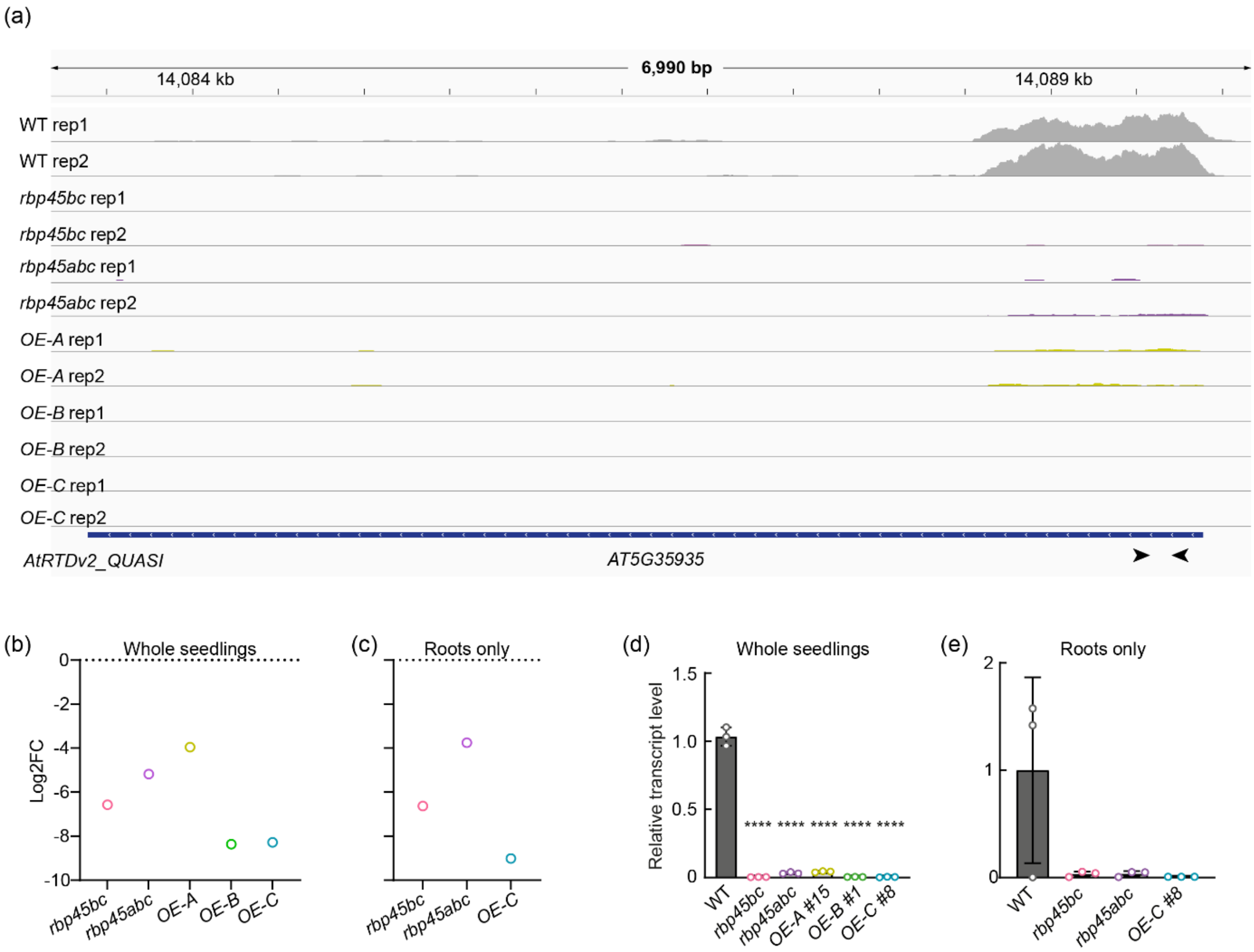

Figure S8: A *Copia-like retrotransposon* is downregulated in all *rbp45* misexpression lines.
(a) Coverage plots from transcriptome sequencing data of 10-d-old *A. thaliana* seedlings of indicated genotypes; all scaled to maximum of 149 reads per position. AtRTDv2_QUASI served as reference genome; the gene model of the *copia-like retrotransposon AT5G35935* is displayed at the bottom with arrows depicting primer binding sites for RT-qPCR analysis shown in (d, e).
(b, c) Change in *AT5G35935* transcript levels in comparison of the RNA-seq data from WT and *rbp45* misexpression lines. The values are negative and indicate downregulation in the mutants relative to WT. Data points represent mean values of two (b) or three (c) biological replicates.
(d, e) RT-qPCR analysis of total transcript level of *AT5G35935* from seedling (d) or root (e) samples. All values are expressed relative to *PP2A* and normalized to the respective mean WT. Mean values (bars), standard deviation (error bars), and individual data points (circles) are depicted. Asterisks indicate significant change compared to WT (one-way ANOVA followed by Dunnett's multiple comparisons test; **** $p < 0.0001$).

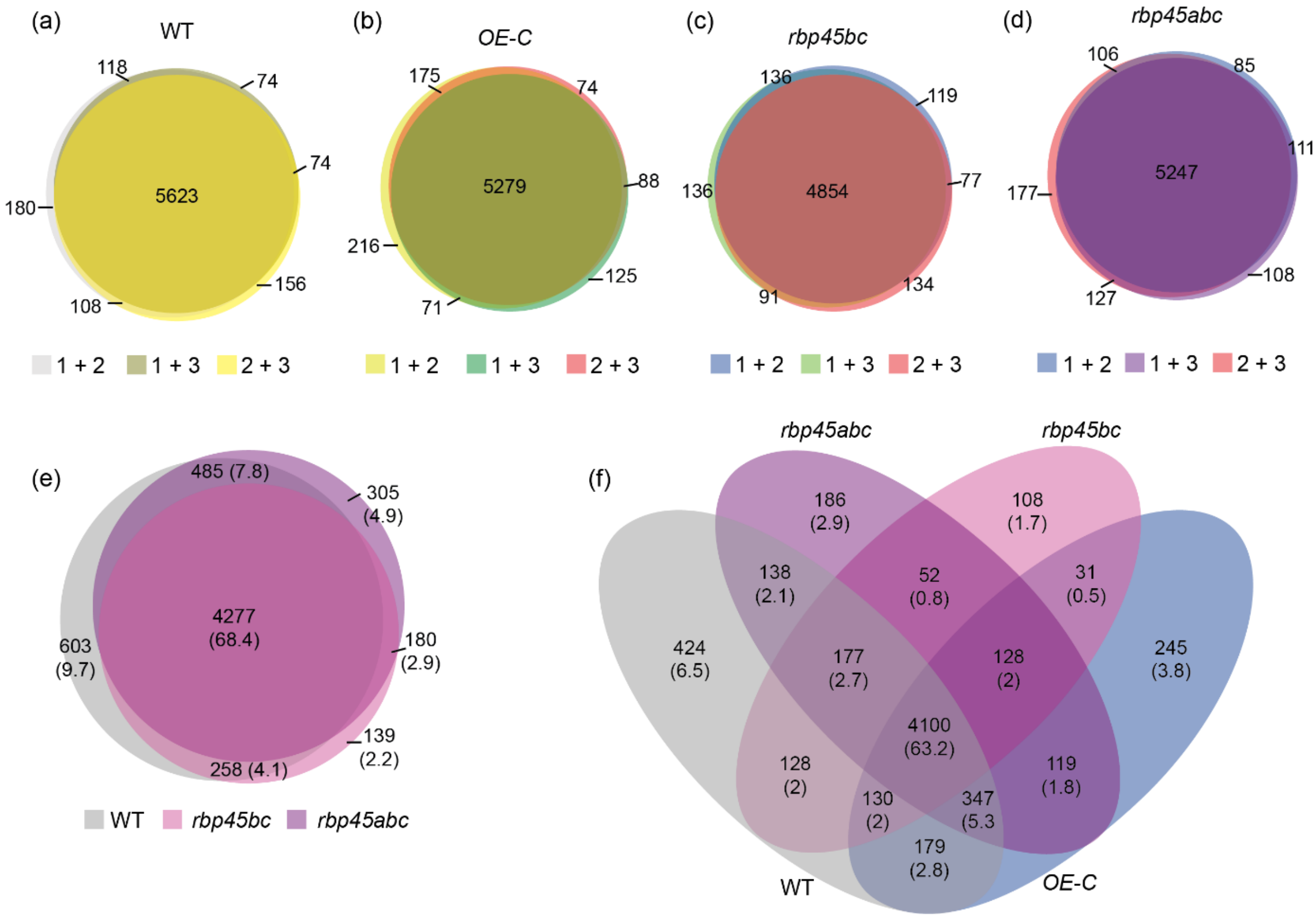

Figure S9: Tissue-specific gene expression in WT and *rbp45* misexpression lines.
(a – d) Comparison of the overlap in DE genes from 10-day-old seedling transcriptome and the 7-day-old root transcriptome for *A. thaliana* WT (a), *RBP45C* overexpression line (b), *rbp45bc* (c), and *rbp45abc* (d). Comparisons include different sets of replicates (indicated by numbers), as for each set of root and whole seedling samples, respectively, 3 and 2 replicates were available, and the analysis required identical number of samples for each sample type. Values in and next to circles depict the number of genes, with numbers in parenthesis providing the percentages.
(e, f) Combination of genotype-specific comparisons from (a -d) of WT and the two *rbp45* knockout lines (d) or in addition the overexpression line *OE-C* (f). For each genotype, only DE genes detected in all replicates were included.

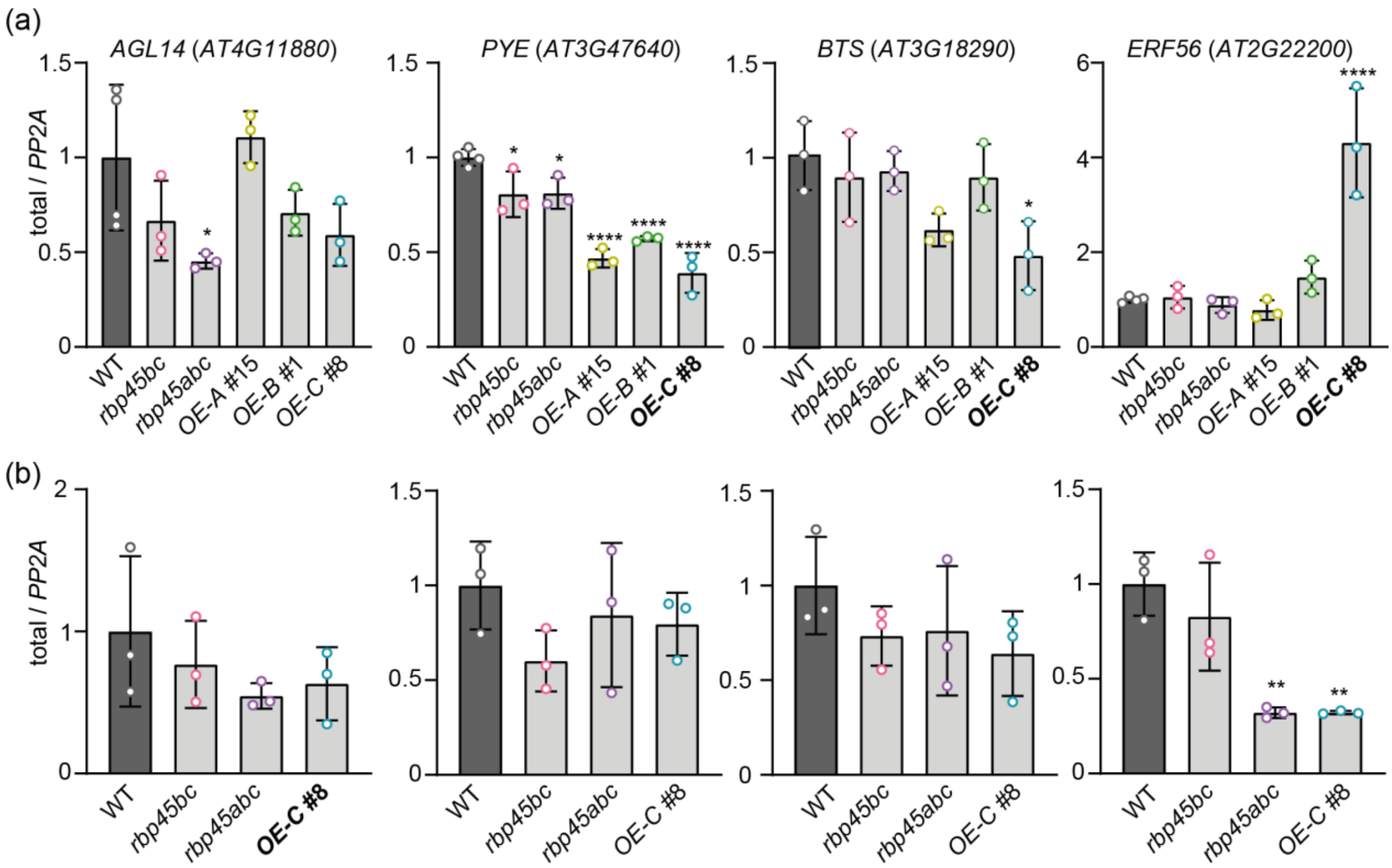

Figure S10*. *Validation of differential gene expression in RBP45 knockout and overexpression lines.

Total transcript levels were determined via RT-qPCR from whole 10-day-old seedlings (a) or roots of 7-day-old seedlings (b). Data were normalized to *PP2A* and relative levels in WT samples were set to 1. Mean values (bars), standard deviations (error bars), and individual data points (circles) are depicted. The sample indicated in bold letters for each gene was found to be differentially expressed compared to WT according to the transcriptome-wide analysis. Asterisks indicate significant change compared to WT (one-way ANOVA followed by Dunnett's multiple comparisons test, *$p < 0.05$, **$p < 0.01$, ****$p < 0.0001$).

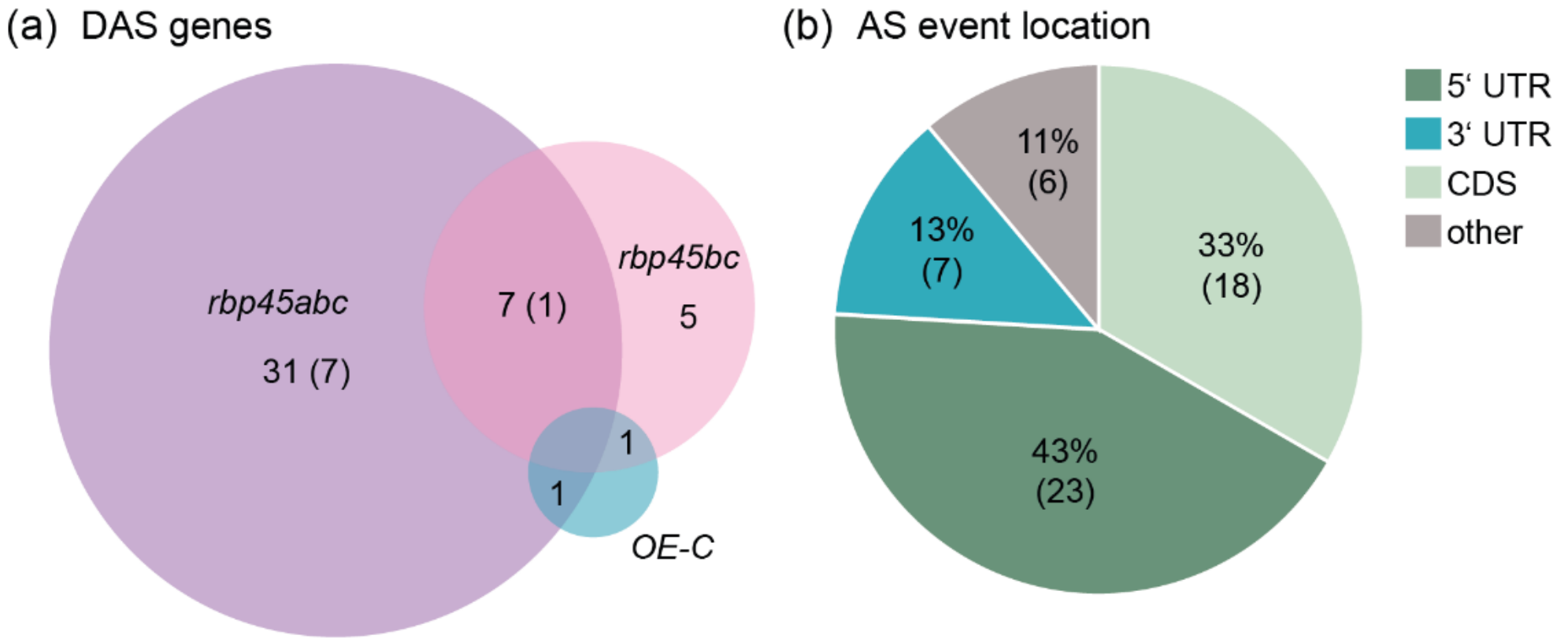

(c)

Location assignment	Number of events
5’ UTR events	5,295
CDS events	21,795
3’ UTR events	1,302
5’ UTR + CDS events	559
CDS + 3’ UTR events	296
5’ UTR + CDS + 3’ UTR events	20

Figure S11: DAS genes and AS event location in root samples of RBP45 misexpression lines.
(a) Venn diagram showing numbers of all significant DAS genes in root samples of 7-day-old *rbp45bc*, *rbp45abc*, and *OE-C* mutants compared to WT. Numbers in parenthesis indicate overlapping genes with seedling data set displayed in Fig. 7d.
(b) Pie chart showing for root samples location of *RBP45*-dependent AS events within the transcripts in proportions and total numbers (in parentheses). For further information and distribution of all events see Fig. 7e.
(c) Location assignments for all events based on AtRTD2-QUASI. Note that the following categories were combined under “other” in Fig. 7e: 5’ UTR + CDS events; CDS + 3’ UTR events; 5’ UTR + CDS + 3’ UTR events, and additional 8,166 events that could not be assigned to any of the basic AS types.

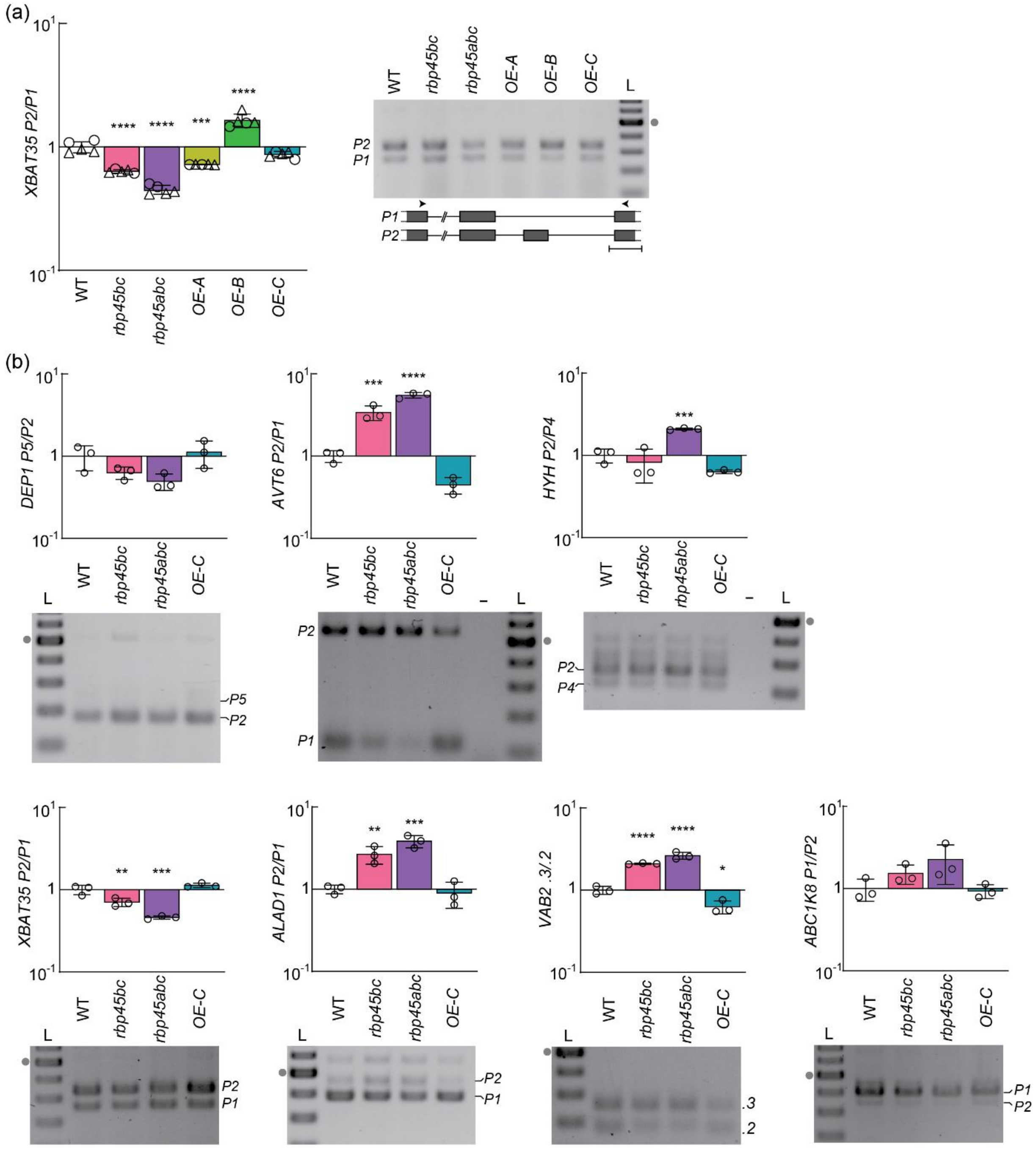

Figure S12. Validation of splicing patterns for candidate genes in RBP45 knockout and overexpression lines.

AS analysis from whole 10-day-old seedlings (a) or roots of 7-day-old seedlings (b) for indicated genes. Bar charts show variant ratios based on Bioanalyzer quantification of co-amplification RT-PCR products. Mean value (bars), standard deviation (error bars), and individual data points (circles: based on samples used for RNA sequencing; triangles: additional replicates) are depicted each; mean AS ratio of WT was set to 1. Asterisks indicate significant change compared to WT (one-way ANOVA followed by Dunnett's multiple comparisons test, *p < 0.05, **p < 0.01, ***p < 0.001, ****p < 0.0001). Representative gel pictures are shown next to or below the bar charts. Models of *XBAT35* splicing variants are shown below the gel in (a) with scale bar corresponding to 100 bp; models for other genes are displayed in Figure 8 and elements are defined in the respective legend. Size ladder (L) for gels consisted of DNAs in 100 bp increments with the strongest band corresponding to 500 bp (marked with gray dot).

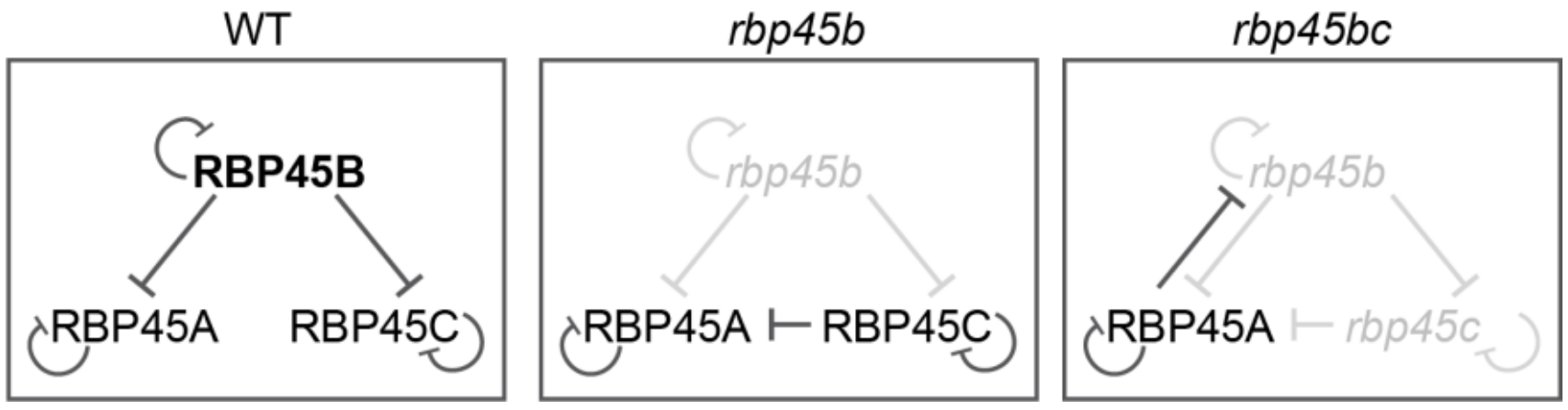

Figure S13. Model of crosstalk and hierarchy in RBP45-mediated splicing regulation. Negative feedback regulation in *A. thaliana* WT, and the knockout mutants *rbp45b* and r*bp45bc*.

Supplemental Methods

Read trimming

BBDuk (v39.38) was executed with the following parameters:

ref=/bbmap/resources/adapters.fa ktrim=r k=23 mink=11 hdist=1 qtrim=r trimq=20 maq=20 ml=20 tpe tbo

Salmon quantification

For the Salmon (v1.10.2) quantification a Salmon index was created from AtRTDv2_QUASI_19April2016.fa and the Arabidopsis genome (TAIR10_chr_all.fas) as decoy sequence.

3D RNA-seq settings

The following settings were used:

Data generation:

- Step 3: tximport method: lengthScaledTPM

Data pre-processing:

- Step 1: CPM cut-off: 4, Sample number cut-off: 1
- Step 4: Data normalization: TMM

3D analysis:

- Step 3: limma-voom pipeline; P-value adjust method: BH; Summarise to DAS gene level p-value: F-test; Adjusted p-value: 0.01; Absolute log_2 FC: 1; Absolute delta PS: 0.1

Determination of AS event locations

- determination of longest ORF per gene from FASTA file of AtRTDv2_QUASI transcriptome (AtRTDv2_QUASI_19April2016.fa)
- extraction of exon coordinates of transcripts from GTF file of AtRTDv2_QUASI transcriptome (AtRTDv2_QUASI_19April2016.gtf)
- transcript with longest ORF set as reference
- calculation of translation start and end position of reference transcript
- check whether first detectable AS event of a transcript isoform locates in 5' UTR, CDS, 3' UTR, or in overlapping regions
- only count AS event, if it is new for the current gene (= deviating coordinate is different from previous deviating coordinates for a given AS event type)
- detection of exitron: end coordinate of exons unequal; end coordinate of reference > end coordinate of isoform; end coordinate of current exon of reference coincides with end coordinate of following exon of isoform
- detection of alternative donor site: end coordinate of exons unequal; start coordinate of following exon of reference coincides with start coordinate of following exon of isoform
- detection of alternative acceptor site: start coordinate of exons unequal; end coordinate of current exon of reference coincides with end coordinate of current exon of isoform
- detection of exon skipping: start coordinate of exons unequal; start coordinate of reference < start coordinate of isoform; start coordinate of the following exon of reference coincides with start coordinate of current exon of isoform

- detection of exon insertion: start coordinates of currently examined exons of reference and isoform are unequal; start coordinate of reference > start coordinate of isoform; start coordinate of current exon of reference coincides with start coordinate of the following exon of isoform
- detection of intron retention: end coordinate of exons unequal; end coordinate of reference < end coordinate of isoform; end coordinate of following exon of reference coincides with end coordinate of current exon of isoform
- detection of mutually exclusive exons: start coordinate of exons unequal; current exon of isoform located in front of current exon of reference or behind it and start coordinates of the following exons of reference and isoform coincide

Outcome of analysis

Event	5'UTR	CDS	3'UTR	5'UTR+CDS	CDS+3'UTR	5'UTR+CDS+3'UTR	Duplicates	Sum
IR	1384	7780	500	0	0	0	2389	12053
AltA	956	7108	249	0	0	0	2824	11137
AltD	1603	3660	113	0	0	0	2365	7741
ER	66	279	20	0	0	0	158	523
ES	83	894	8	81	6	0	501	1573
CI	1202	2056	412	477	289	20	2243	6699
MXE	1	18	0	1	1	0	12	33
Sum	5295	21795	1302	559	296	20	10492	-

Sum of transcripts with unresolved AS events: 8166

Python scripts

See https://github.com/Christoph-cloud-ux/locate_AS
